# Cancer-associated fibroblasts and immune escape-related genes serve as biological markers for the prognosis of colorectal cancer

**DOI:** 10.1186/s41065-026-00676-9

**Published:** 2026-04-07

**Authors:** Xia Liu, Aorong Shi, Bai Dai, Yue Chang, Fen Yun, Huan Guan, Rina Sha, Yongfeng Jia

**Affiliations:** 1 School of Public Health, Shanxi Medical University, Taiyuan, Shanxi China; 2 Inner Mongolia Medical University, Hohhot, Inner Mongolia China; 3 Affiliated Hospital of Inner Mongolia Medical University, Hohhot, Inner Mongolia China

**Keywords:** Colorectal Cancer, Cancer-associated fibroblasts, Immune escape, Bioinformatics analysis, Hub gene, Prognostic model

## Abstract

**Background:**

Colorectal cancer (CRC) is a common malignant tumor with a steadily rising incidence rate, and it is often diagnosed at an advanced stage. Immunotherapy has emerged as a promising alternative to surgical resection. Cancer-associated fibroblasts (CAFs) play a critical role in the tumor microenvironment (TME) and are closely associated with tumor immune evasion. This study aims to identify genes associated with CAFs and immune evasion in CRC, and to explore their underlying mechanisms and potential clinical applications in CRC.

**Methods:**

Based on CRC transcriptomic data from TCGA and GEO (GSE39582), this study identified differentially expressed genes associated with tumor-associated fibroblasts and immune evasion (CAFIERDEGs) and performed GO/KEGG functional enrichment analysis on them. A prognostic risk model was constructed based on CAFIERDEGs, followed by survival analysis and validation. Additionally, key genes were identified through the construction of a protein-protein interaction (PPI) network, and differences in immune checkpoint expression, immune cell infiltration, and immunotherapy response were systematically evaluated across different risk groups. Finally, the key genes were experimentally validated using single-cell RNA sequencing, immunohistochemistry, and qPCR.

**Results:**

Five hub genes (SOX2, CXCL11, SPP1, APOE, and CXCL8) were screened. These genes are involved in the regulation of the immune response, cytokine activity, and the Toll-like receptor signaling pathway. CRC patients were classified into two distinct molecular subtypes based on the expression profiles of these genes. The prognostic risk model revealed significant differences in overall survival rates. The tumor immune dysfunction and rejection (TIDE) score was significantly lower in the low-risk group. The tumor mutational burden (TMB) was higher, and the expression of most immune checkpoint genes increased. This indicates a stronger potential reactivity to immunotherapy. Experiments confirmed that the expressions of CXCL8, CXCL11, and SPP1 were significantly upregulated in CAFs, while APOE and SOX2 expressions were significantly downregulated.

**Conclusion:**

This study identified five CAFIERGs and constructed a prognostic model for CRC by integrating CAFs and immune escape-related genes. This model can predict the immunotherapy response and prognosis of CRC patients. These findings offer potential therapeutic targets for improving prognostic evaluation and guiding immunotherapy strategies in CRC patients.

## Introduction

CRC is a common malignant neoplasm characterized by a high incidence and mortality rate. Challenges in its treatment primarily arise from difficulties in early detection, tumor heterogeneity, biological propensity for metastasis and recurrence, and eventual drug resistance. Additionally, the immunosuppressive tumor microenvironment facilitates the immune escape of cancer cells, thereby limiting the efficacy of immunotherapy [1]. CAFs are recognized as the primary stromal cell type within the TME of CRC. Via diverse mechanisms, CAFs promote the development of an immunosuppressive TME, significantly reducing the efficacy of immunotherapeutic approaches [2]. Following activation, CAFs proliferate extensively and secrete large quantities of extracellular matrix (ECM) proteins, such as collagen and fibronectin. This contributes to the formation of a dense fibrotic stroma. This fibrotic structure acts as a physical barrier that impedes the infiltration of immune cells, such as cytotoxic T lymphocytes (CTLs), into the tumor core. Consequently, these immune cells cannot effectively recognize and target cancer cells, limiting the efficacy of immunotherapeutic agents [3, 4]. Second, CAFs secrete cytokines and chemokines that can directly inhibit immune cell function or recruit immunosuppressive cells. Among these factors, TGF-β is recognized as the most critical and potent immunosuppressive molecule [5]. TGF-β can suppress CTL and NK cell activity while promoting the development and functional activity of populations such as Tregs and MDSCs [6]. CXCL12 can sequester T cells within the ECM via its receptor, CXCR4, thereby preventing them from migrating toward cancer cells. Additionally, it recruits immunosuppressive cell populations [7]. IL-6, VEGF, PGE2, and other cytokines contribute to the formation of an immunosuppressive inflammatory environment [8]. CAFs compete with cancer cells for essential nutrients, such as glucose and amino acids, resulting in nutrient depletion and increased acidity within the TME. Activated T cells rely on these nutrients to carry out their effector functions. This phenomenon, referred to as metabolic competition, can impair T cell activity, leading to functional exhaustion and reduced capacity to effectively eliminate tumor cells [9]. CAFs can highly express immune checkpoint proteins such as PD-L1 and PD-L2 [10]. When T cells attempt to attack CAFs, these proteins bind to PD-1 receptors on T cells’ surfaces, transmitting a “don’t eat me” signal. This signal indirectly shields adjacent cancer cells from immune-mediated destruction [11].

Advancements in high-throughput sequencing technology have created unprecedented opportunities to investigate the molecular characteristics of CRC [12]. Machine learning–based predictive models are transforming approaches to evaluating and managing prognosis in CRC. These models integrate and analyze large-scale, multidimensional datasets to offer more accurate and personalized prognostic predictions than traditional methods, thereby providing robust support for clinical decision-making [13]. In this study, we performed a comprehensive analysis of publicly available datasets to identify genes associated with CRC-CAFs and immune escape mechanisms. We examined their differential expression patterns, developed a prognostic risk model, and analyzed their relationship with immune cell infiltration. Our findings provide valuable insight into the functional roles and clinical relevance of CAFIERGs in CRC and could inform the development of novel therapeutic strategies.

## Materials and methods

### Data download

The CRC dataset (TCGA-COADREAD) was analyzed using the TCGAbiolinks R package [14] (v2.30.0) for data acquisition from TCGA (https://portal.gdc.cancer.gov/). After excluding the samples with incomplete clinical records, we obtained raw count sequencing data for 647 CRC specimens (designated as CRC group). These data were subsequently normalized to FPKM values (Fragments Per Kilobase per Million) for standardized comparison. Complementary clinical metadata and 51 adjacent normal tissue samples (control group) were retrieved through the UCSC Xena platform [15] (https://xena.ucsc.edu/). The combined dataset containing both CRC and control samples in count format underwent comprehensive bioinformatics processing. The comprehensive clinical features were presented in Table 1.

**Table 1:** Patient characteristics of CRC patients in the TCGA-COADREAD datasets

Characteristics	overall
OS, n (%)	
Alive	497 (79.9%)
Dead	125 (20.1%)
Age, n (%)	
< 65	251 (40.4%)
≥ 65	371 (59.6%)
Gender, n (%)	
Female	287 (46.1%)
MALE	335 (53.9%)
Stage, n (%)	
Stage I	109 (18.1%)
Stage III	179 (29.8%)
Stage II	226 (37.6%)
Stage IV	87 (14.5%)

The CRC dataset (GSE39582) was retrieved from the GEO database [16] (https://www.ncbi.nlm.nih.gov/geo/) using the R package GEOquery [17]. All samples in the GSE39582 dataset [18] were derived from Homo sapiens, sourced from colorectal frozen tissues, and generated using the GPL570 microarray platform. Detailed information was listed in Table 2. The dataset comprises 566 CRC samples and 19 control samples, all of which were included in the present study.

**Table 2:** GEO microarray chip information

Platform	GSE39582
	GPL570
Species	Homo sapiens
Tissue	Frozen tissue of colorectal
Samples in CRC group	566
Samples in Control group	19
Reference	^[5]^

To construct sets of cancer-associated fibroblast-related genes (CAFRGs) and immune escape-related genes (IERGs), searches were conducted in the GeneCards database [19](https://www.genecards.org/) using the keywords “Cancer-associated Fibroblast [20]” and “Immune Escape [21],” respectively. Only protein-coding genes were retained for subsequent analysis. During the GeneCards search, the relevance score threshold was set to greater than 1. Concurrently, a literature search was conducted in the PubMed database using the same keywords, prioritizing relevant articles published within the past three years in high-impact journals. Inclusion criteria were: the content of the literature must be related to CAFs or immune escape, and it must explicitly report the names of relevant genes or sets of gene characteristics; duplicate reports, articles lacking clear gene information, or those with weak relevance to the research topic were excluded. After merging the database search results with the literature review findings, duplicate entries were removed and gene symbols were standardized. This process ultimately yielded a set of 758 CAFRGs and 891 IERGs, identifying 76 CAFIERGs (see Supplementary Table S1 for details).

### Batch effect correction

To mitigate the effects of non-biological variations introduced by different experimental platforms and technologies, a systematic correction for batch effects was performed after integrating gene expression data from the TCGA and GEO databases. The procedure involved the following steps: RNA-sequencing data from the TCGA-COADREAD cohort were converted uniformly to FPKM values and then log₂ transformed. Meanwhile, the microarray data from the GSE39582 dataset underwent background adjustment, log₂ transformation and quantile normalisation. The expression matrices from the two datasets were then merged. Batch effect adjustment was then carried out using the ComBat function from the R package sva, specifying the data source (i.e. database) as the batch variable. This corrected, integrated expression matrix was used for all subsequent analyses, including identifying differentially expressed genes, performing molecular subtyping, assessing immune cell infiltration and constructing the prognostic risk model. This ensured the reliability and accuracy of the findings.

### CRC-related fibroblasts and immune escape-related differentially expressed genes

Based on the sample classification in the CRC dataset (TCGA-COADREAD), samples were categorized into the CRC group and the control group. Gene expression differences between the CRC and control groups were analyzed using the R package limma [22] (Version 3.58.1). Volcano plots were generated using the R package ggplot2 (Version 3.4.4) to visualize the results of the differential expression analysis. The threshold for differentially expressed genes (DEGs) was set to|logFC| > 1 & adj.P.Val < 0.05. Genes with logFC > 1 & adj.P.Val < 0.05 were considered upregulated, while those with logFC < -1 & adj.P.Val < 0.05 were considered downregulated. To obtain CAFIERDEGs, all DEGs with |logFC| > 1 & adj.P.Val < 0.05 obtained by differential analysis in the CRC dataset (TCGA-COADREAD) were interposed with CAFIERGs, resulting in a Venn diagram being plotted. CAFIERDEGs were obtained. At the same time, to further evaluate the prognostic significance of CAFIERDEGs in CRC, a univariate Cox regression analysis was performed, according to the intersection results, all variables with a p-value < 0.10 in the univariate analyses were included in subsequent analyses. A heatmap illustrating the screened CAFIERDEGs was generated using the R package pheatmap (Version 1.0.12), while chromosomal localization was visualized with the R package RCircos [23] (Version 1.2.2).

### GO and pathway (KEGG) enrichment analysis

Gene Ontology (GO) analysis [24] is a commonly employed method for conducting in-depth functional annotation studies, covering three main domains: Biological Process (BP), Cellular Component (CC), and Molecular Function (MF). The KEGG [25] represents a fundamental biological resource containing comprehensive data on genomic networks, metabolic pathways, disease mechanisms, and pharmaceutical compounds. The analysis was conducted using clusterProfiler [26] (Version 4.10.0) in R to perform functional enrichment assessments of identified CAFIERDEGs through both GO classification and KEGG pathway examination, with significance thresholds set at adjusted p-values < 0.05 and false discovery rate (q-value) < 0.25 while employing the Benjamini-Hochberg (BH) procedure for multiple testing correction.

### Construction of CRC subtypes

Consensus clustering [27] methodology employs repeated subsampling to generate measurement variability, enabling quantitative assessment of cluster robustness and optimal parameter determination. This analytical approach was implemented via the R package ConsensusClusterPlus [28], specifically applied to delineate molecular subtypes within colorectal cancer specimens from the TCGA-COADREAD cohort. In this process, the number of clusters was set from 2 to 9, and 80% of the total samples were sampled 50 times with clusterAlg ="pam” and distance = “euclidean”. Subsequently, the expression differences among various disease subtypes in the CRC dataset (TCGA-COADREAD) were analyzed by using an expression value heatmap, and these differences were subsequently confirmed using a group comparison visualization.

### Gene set enrichment analysis (GSEA) among CRC subtypes

The CRC dataset (TCGA-COADREAD) was divided into CRC subtype one (Cluster 1) group and CRC subtype two (Cluster 2) group according to the consensus clustering results, and the R package limma was used to analyze the differences. The threshold value of |logFC| > 1 and adj.P.Val < 0.05 was set as the DEGs. Genes with logFC > 1 & adj.P.Val < 0.05 indicated upregulated genes, while genes with logFC < -1 & adj.P.Val < 0.05 indicated downregulated genes. A volcano map was drawn using the differential analysis results through the R package ggplot2, and the heatmap was drawn using deg through the R package pheatmap.

GSEA [29] evaluates the distribution pattern of genes within a predefined gene set across an expression profile ranked by phenotypic correlation, aiming to identify their potential contribution to specific phenotypic traits. Firstly, we ranked the genes in the CRC dataset (TCGA-COADREAD) based on the logFC values between CRC subtype one (Cluster 1) and subtype two (Cluster 2). GSEA was conducted on the entire set of genes from the CRC dataset (TCGA-COADREAD) using the R package clusterProfiler. GSEA was conducted using filtering criteria of *p* < 0.05, FDR value (q-value) < 0.25, and applying Benjamini-Hochberg for p-value correction.

### Construction of prognostic risk model and clinical manifestations of CRC

In the CRC(TCGA-COADREAD) cohort, we constructed a predictive risk assessment model using the LASSO method. Based on the prognosis-related genes identified in a preliminary screening, we performed LASSO Cox regression analysis using the R package glmnet (Version 4.1-8), with the parameter family set to “cox,” and determined the optimal penalty parameter lambda through 10-fold cross-validation. The optimal lambda was determined using lambda.min, i.e., the lambda value corresponding to the minimum partial likelihood error. Under these parameters, key genes were selected for inclusion in subsequent model development.

### Validation of prognostic risk models for CRC

In order to further verify the accuracy and discrimination of the prognostic risk model for CRC, the LASSO risk score of the CRC dataset (GSE39582) was calculated based on the expression of key genes of CRC in the TCGA COADREAD dataset and the LASSO coefficient of the CRC prognostic risk model. According to the median value of LASSO risk score, the CRC samples from the TCGA COADREAD dataset were classified into high-risk and low-risk subgroups based on risk stratification. The risk score was calculated using the following formula:

$$\:\mathrm{R}\mathrm{i}\mathrm{s}\mathrm{k}Score\:=\:\sum\limits_i\:Coefficient\:\left(gene_i\right)\mathrm{*}mRNA\:Expression\:\left(gene_i\right)$$

To analyze the difference in overall survival (OS) between high-risk and low-risk groups of CRC in the CRC dataset (TCGA-COADREAD), KM curves [30] analysis was performed using the R package survival [31] (Version 3.5-7), and a KM curve was drawn based on LASSO risk score. Time-dependent Receiver Operating Characteristic (ROC) curves [32] serve as a visual analytical tool for evaluating and selecting the optimal model based on predictive performance over time. The R package survivalROC (Version 1.0.3.1) was employed to generate ROC curves and compute the corresponding Area Under the Curve (AUC) using the LASSO-derived risk score and OS data. To assess the predictive performance for 1-, 3-, and 5-year survival outcomes of CRC samples from the GSE39582 dataset, we also computed the corresponding AUC values. The AUC values of the ROC curves typically ranged between 0.5 and 1, with values approaching 1 reflecting higher diagnostic accuracy and better predictive capability.

The calibration curves were implemented to illustrate the alignment between observed probabilities and model-predicted outcomes across various scenarios, enabling quantitative assessment of predictive accuracy through systematic comparison of empirical data versus theoretical estimations. Using the R package ggDCA [33] (Version 1.1), we generated a decision curve analysis (DCA) plot based on the LASSO risk score, integrating clinical variables into a multivariable Cox regression framework to evaluate the predictive accuracy and discriminative performance of the colorectal cancer prognostic model.

### Protein-protein interaction (PPI) network and hub gene screening

The PPI network consists of interacting proteins that collectively participate in various biological processes, including signal transduction, regulation of gene expression, energy and metabolic pathways, as well as cell cycle control. The STRTING database [34] (https://string-db.org/) is a comprehensive knowledge base and prediction platform for PPI, used for constructing, analyzing and visualizing functional association networks between proteins. In this study, the STRING database was utilized to construct a PPI network centered on key genes, applying a medium confidence interaction score threshold of > 0.400. This threshold helped identify functionally relevant interactions, where densely interconnected subnetworks may correspond to protein complexes and potentially share specific biological roles. Genes exhibiting interactions with multiple partners in the PPI network were identified and designated as hub genes for further analysis. The CytoHubba [35] plugin in Cytoscape [36] was used to implement five centrality algorithms: Maximum Clique Centrality (MCC), Degree, Maximum Neighborhood Component (MNC), Edge Percolated Component (EPC), and Closeness [37]. These algorithms were applied to compute centrality scores for genes within the PPI network, which were then ranked to identify the most central and potentially functionally significant nodes.

### Immune infiltration analysis of high-risk and low-risk groups

We adopted the CIBERSORT [38] algorithm and combined it with the characteristic gene matrix of immune cells to analyze the infiltration of immune cells in the CRC dataset (TCGA-COADREAD). To ensure the specificity and reliability of the analysis, we filtered out the data with an immune cell enrichment score no greater than zero, and ultimately obtained the statistically significant immune cell infiltration matrix results in this dataset. Inter-group comparison plots depicting the differences in immune cell composition between high-risk and low-risk groups in the CRC dataset (TCGA-COADREAD) were generated using the R package ggplot2 (Version 3.4.4). The Spearman algorithm was then applied to compute pairwise correlations among immune cell types, and the results were visualized as a correlation heatmap using the R package heatmap (Version 1.0.12), displaying the interrelationships between immune cells in the dataset. Furthermore, the Spearman algorithm was used to assess the relationships between hub genes and immune cells, and the resulting associations were visualized through a bubble plot created with the R package ggplot2.

### Immunotherapy analysis of hub genes

In order to obtain the TIDE immune scoring results for CRC in the CRC dataset (TCGA-COADREAD), an analysis was performed on the expression matrix based on the CRC dataset (TCGA-COADREAD) using the TIDE web platform [39, 40] (http://tide.dfci.harvard.edu). Based on the TIDE immunoscore analysis results, we calculated the difference in TIDE immunoscore between the high-risk and low-risk groups of CRC by the Mann-Whitney U Test (Wilcoxon Rank Sum Test) in the CRC dataset (TCGA-COADREAD).Then, we downloaded TMB data and microsatellite instability (MSI) data from the cBioPortal database [41] (https://www.cbioportal.org/). Finally, the mutation counts of the high-risk and low-risk groups in the CRC dataset (TCGA-COADREAD) were calculated using the Mann-Whitney U Test, and the differences between the groups were assessed using the TMB score.

Immunogenicity refers to the ability of an antigen or its epitopes to induce humoral and/or cell-mediated immune responses through recognition by antigen receptors on T cells and B cells. This principle of immunogenicity can be referred to as an immunogen. With machine learning, immunogenicity can be estimated and quantified. The Cancer Immunome Atlas (TCIA) database [42] (https://tcia.at/home) offers immunophenotypic scores (IPS) across 20 cancer types and has demonstrated potential in predicting responses to immune checkpoint molecules such as CTLA-4 and PD-1. In this study, IPS data for colorectal cancer (CRC) samples were retrieved from the TCIA’s CRC cohort (TCGA-COADREAD). To compare immune profiles between risk groups, a group-wise visualization of IPS was generated using the R package ggplot2 (Version 3.4.4), enabling an assessment of differences in various IPS components between high-risk and low-risk patients.

Immune checkpoint blockade therapy has brought significant clinical benefits against several solid malignancies. Fifty immune checkpoint-associated genes were collected based on the work of Fang Jun et al. [43]. The expression differences between the high-risk group and the low-risk group were analyzed and then displayed in a group comparison chart.

### In vitro co-culture experiments

The mouse CRC cells (CT26.WT), as well as the mouse fibroblasts (NIH/3T3), were purchased from Procell Life Science & Technology Co., Ltd. (Wuhan, China). The CT26.WT cells were cultured in an RPMI 1640 medium containing 10% FBS and 1% penicillin-streptomycin. The NIH/3T3 cells were cultured in DMEM medium containing 10% FBS and 1% penicillin–streptomycin. The NIH/3T3 and CT26.WT cells were co-cultured, with the cells separated by a 0.4 μm Transwell™ insert. The CT26.WT cells were inoculated in the upper chamber of a 6-well plate at a density of approximately 9 × 10³ cells per well with 1.5 mL of culture medium. The lower chamber contained 2 × 10⁴ NIH/3T3 cells per well with 2.5 mL of culture medium. The medium was changed daily, and the culture was conducted for four days. Then, the cells in the lower chamber were collected for subsequent experiments. All cells were cultured at 37 °C in a humidified atmosphere containing 5% CO₂.

### RNA extraction and qRT-PCR

According to the manufacturer’s instructions, total RNA was extracted from NIH/3T3 cells using TRI-ZOL (lot no. A054250520, Beyotime). Then, a one-step RT kit (TransGen Biotech, Beijing) was used to reverse transcribe the RNA into cDNA. The qRT-PCR reaction was conducted using an Archimed Real-Time PCR system. PPIA was used as an internal control with the following primers: M-PPIA-F: TGCCAAGACTGAATGGCTGG and M-PPIA-R: AAAACGCTCCATGGCTTCG. The qRT-PCR reactions were carried out using an AQ601-02-V2 qRT-PCR kit (TransGen Biotech) according to the manufacturer’s instructions, and the experiment was repeated three times.

### Immunohistochemical verification

The expression and localization of hub gene-encoded proteins in CRC tissues were verified using the online resource The Human Protein Atlas (HPA) database. To perform the analysis, the official HPA website (https://www.proteinatlas.org) was accessed. The target gene names were entered individually into a search box, leading to their respective gene summary pages. Subsequently, the “Cancer” section was selected, and “Colorectal Cancer” was chosen for further analysis. Immunohistochemical staining images of colorectal cancer tissue microarrays provided by HPA were then evaluated.

### Single-cell sequencing of CRC FFPE samples

FFPE samples from CRC and the corresponding adjacent normal tissues were collected from five patients diagnosed in June and July of 2024 at the Department of Pathology at the Affiliated Hospital of Inner Mongolia Medical University. These samples were used for single-cell sequencing. The inclusion criteria were as follows: (1) histologically confirmed primary CRC based on postoperative pathological examination and (2) no prior history of radiotherapy, chemotherapy, targeted therapy, or immunotherapy before surgery. Use of the human CRC tissues was approved by the Medical Ethics Committee of Inner Mongolia Medical University (Ethics Approval Number YKD202403015). Single-Cell Nuclei Preparation and RNA-Seq Library Construction from FFPE Tissues. FFPE tissue scrolls (50 μm) were deparaffinized, rehydrated, and homogenized in cold lysis buffer using a Dounce homogenizer. The number of nuclei was estimated using the SeekMate Tinitan Fluorescence Cell Counter. Nuclear RNA was extracted with the SeekOne^®^ DD Single Cell Decrosslinking Kit, and its integrity was assessed on the Agilent 4200 TapeStation. Qualified nuclei were pre-decrosslinked, re-fixed, recounted, and adjusted to a concentration of 1,500–2,000 nuclei/µl.For single-cell RNA-seq, libraries were prepared using the SeekOne^®^ DD FFPE Single Cell Transcriptome-seq Kit. Reverse transcription was performed on 50,000–100,000 nuclei with 15 cycles of annealing. Following washing, the nuclei were combined with ligation reagents, encapsulated into emulsion droplets using the SeekOne^®^ Digital Droplet System, and barcoded cDNA was generated. The cDNA was purified, amplified with index PCR, and the final libraries were sequenced on the Illumina NovaSeq 6000 with a PE150 read length.

### Statistical analysis

All data processing and analyses described in this study were performed using R software (Version 4.3.1). To compare continuous variables between two groups, we applied the independent Student’s t-test to evaluate statistical significance in cases of normally distributed data. We employed the Mann-Whitney U test (also known as the Wilcoxon rank sum test) to assess differences in variables that did not follow a normal distribution. The Kruskal-Wallis test was used to compare three or more groups. Spearman’s correlation analysis was used to calculate the correlation coefficient between different molecules., and adj. *p* < 0.05 was considered statistically significant.

## Results

###  Technology roadmap(Fig.1 )

**Fig. 1:**
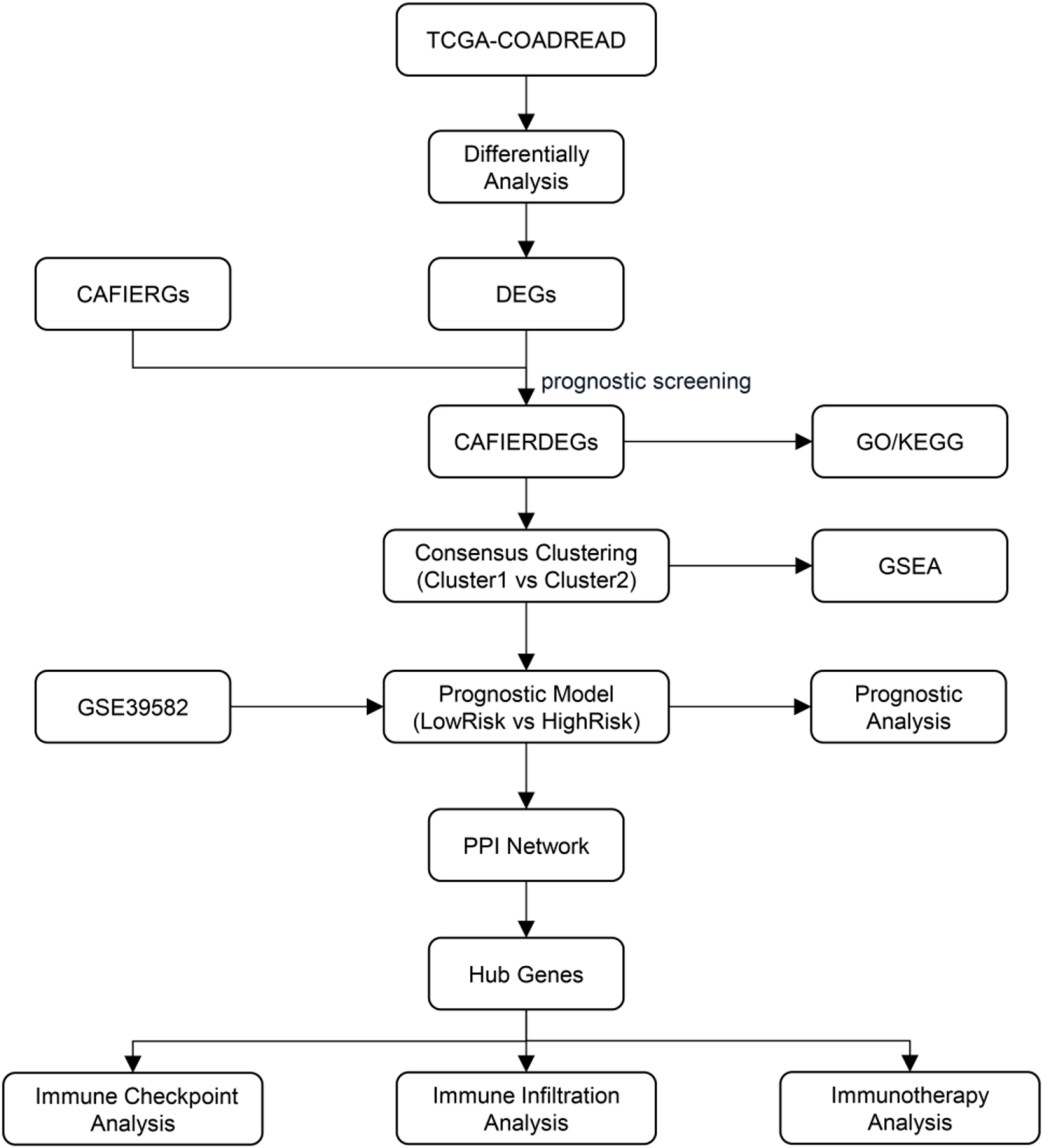
Technology roadmap

### CRC related fibroblasts and immune escape related differentially expressed genes

**Fig. 2:**
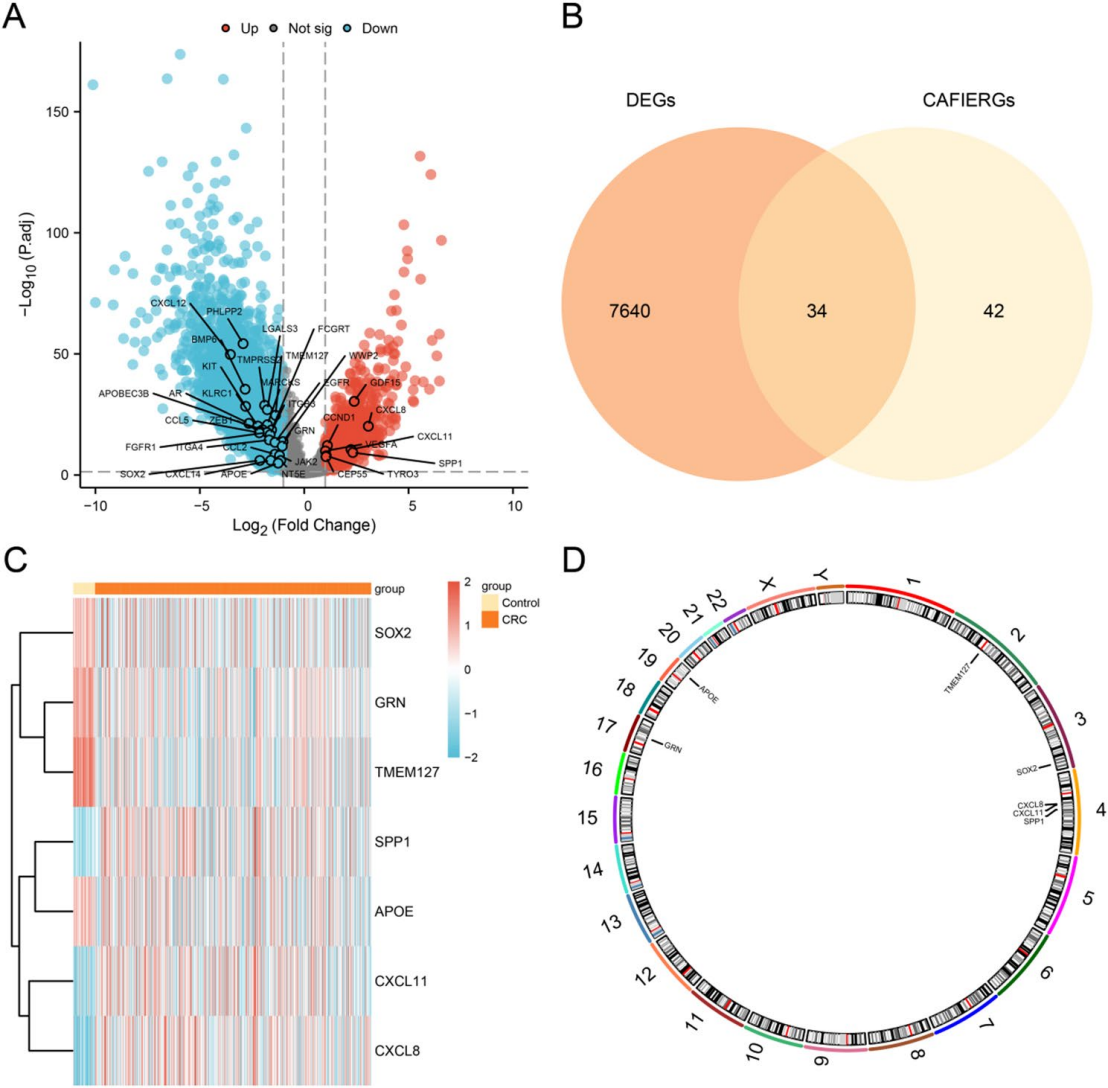
Differential gene expression analysis in the CRC dataset (TCGA-COADREAD). **A**. Volcano plot illustrating the differential expression analysis between the CRC and control groups, with 34 CAFIERDEGs highlighted as marker genes. **B** Venn diagram depicting the overlap between DEGs and CAFIERGs. **C** Heatmap displaying the expression patterns of seven prognostic-associated CAFIERDEGs. **D** Chromosomal localization map showing the genomic positions of the seven prognosis-related CAFIERDEGs

The analysis revealed that in the CRC dataset (TCGA-COADREAD), 7,674 DEGs satisfied the criteria of |log₂FC| > 1 and adjusted p-value < 0.05, comprising 1,193 upregulated genes (log₂FC > 1, *p* < 0.05) and 6,481 downregulated genes (log₂FC < -1, *p* < 0.05). A volcano plot (Fig. 2A) was generated to visualize the results of this differential expression analysis.

To obtain CAFIERDEGs, DEGs with logFC > 1 & *p* < 0.05 were intersected with CAFIERGs, and a Venn diagram (Fig. 2B) was plotted. A total of 34 CAFIERDEGs were obtained, including PHLPP2, CXCL12, BMP6, GDF15, TMPRSS2, KIT, LGALS3, TMEM127, AR, MARCKS, KLRC1, CXCL8, and PHLPP2. APOBEC3B, FCGRT, ZEB1, FGFR1, CCL5, ITGB3, ITGA4, WWP2, EGFR, CCND1, GRN, CXCL11, VEGFA, SPP1, CCL2, JAK2, TYRO3, CEP55, CXCL14, SOX2, NT5E and APOE. To further evaluate the prognostic significance of the 34 CAFIERDEGs in CRC, univariate Cox regression analysis was performed. Based on the intersection results, all genes with a p-value < 0.10 in the initial univariate assessment were selected for inclusion in subsequent multivariate analyses. A total of seven prognostic-related CAFIERDEGs were obtained: GRN, SPP1, APOE, TMEM127, CXCL11, CXCL8, and SOX2. The specific information is shown in Table 3. Based on the intersection findings, the expression variations of seven CAFIERDEGs across different sample groups in the CRC dataset (TCGA-COADREAD) were examined, and a heatmap was generated using the R package pheatmap to visualize the differential expression patterns of these genes (Fig. 2C). Finally, the chromosomal locations of these seven CAFIERDEGs were analyzed using the R package RCircos, and a chromosome localization map was generated (Fig. 2D). The genes were distributed across chromosomes 2, 3, 4, 17, and 19: TMEM127 was mapped to chromosome 2; SOX2 to chromosome 3; CXCL8, CXCL11, and SPP1 to chromosome 4; GRN to chromosome 17; and APOE to chromosome 19.

**Table 3:** Prognostic gene screening results in TCGA-COADREAD

gene_name	hr	pvalue
GRN	1.280004	0.059215
PHLPP2	0.854491	0.453648
FGFR1	1.075722	0.565981
TYRO3	1.207710	0.209149
JAK2	0.868759	0.327648
FCGRT	1.012043	0.893083
CXCL12	1.086862	0.368863
CCL2	1.044247	0.588725
CCND1	1.002046	0.988621
VEGFA	1.168878	0.255936
ITGA4	0.846580	0.305817
SPP1	1.074389	0.080539
APOE	1.110949	0.035740
GDF15	0.951243	0.501310
LGALS3	0.987971	0.913298
KLRC1	0.787959	0.611001
NT5E	1.037500	0.634951
TMEM127	2.059785	0.005987
CEP55	0.846906	0.160028
CXCL14	0.939954	0.163929
EGFR	1.097683	0.526402
ZEB1	0.983224	0.886019
BMP6	1.138512	0.450181
KIT	0.995861	0.972067
AR	1.289753	0.362900
CXCL11	0.885004	0.043601
CXCL8	0.911507	0.056452
APOBEC3B	0.915735	0.438574
SOX2	1.117742	0.097889
TMPRSS2	1.134343	0.281035
WWP2	1.004976	0.982878
ITGB3	0.827021	0.311588
CCL5	0.994002	0.932792
MARCKS	0.955598	0.713253

### Gene ontology (GO) and pathway (KEGG) enrichment analysis

**Fig. 3:**
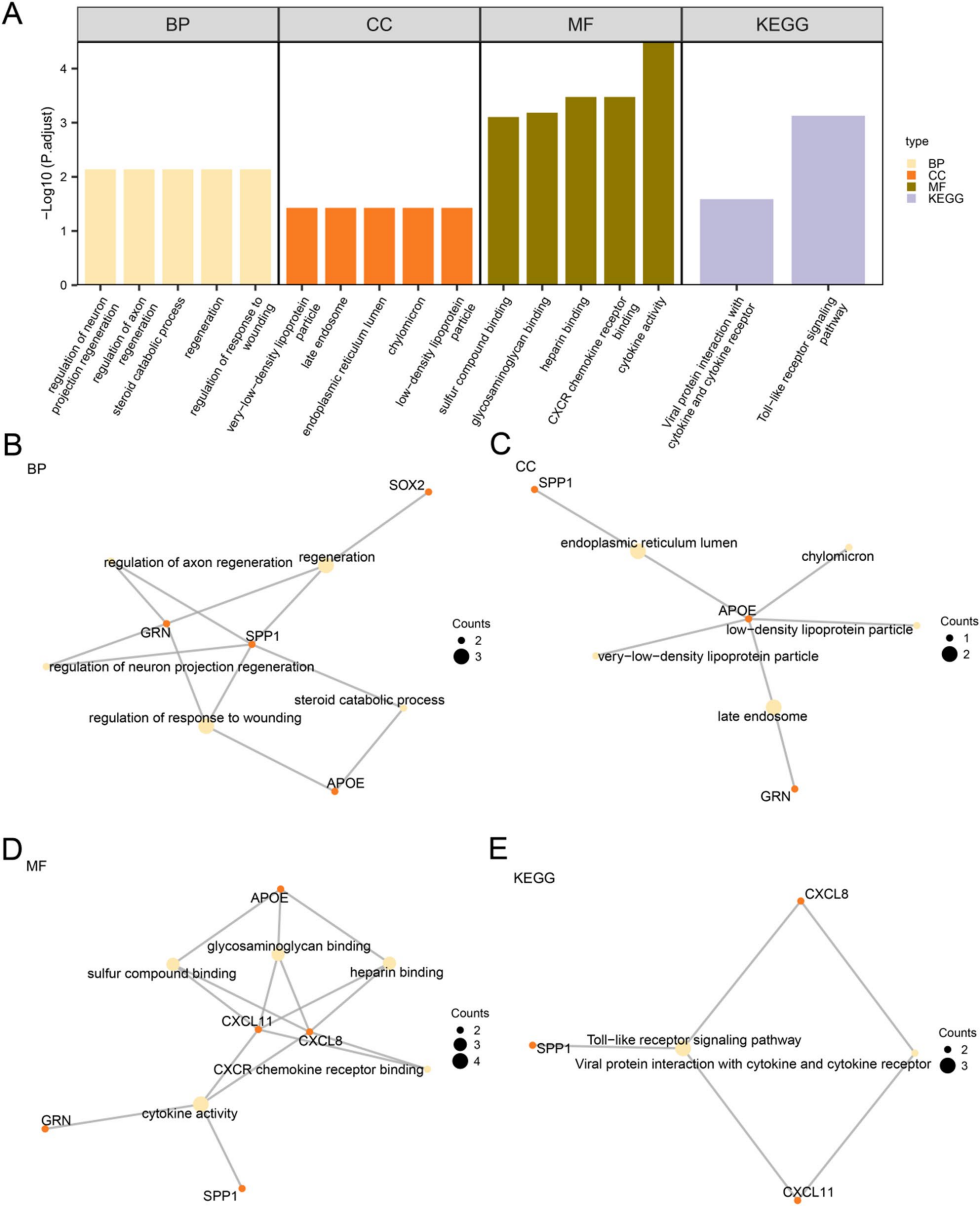
GO and KEGG enrichment analysis. **A** Bar chart of GO and KEGG enrichment analysis results of seven CAFIERDEGs : BP (**B**), CC (**C**), MF (**D**) and KEGG (**E**)

GO and pathway enrichment analysis were used to further explore the relationship between biological process (BP), cellular component (CC), molecular function (MF), and biological pathway (KEGG) of seven CAFIERDEGs and CRC. GO and KEGG enrichment analyses were performed using the seven CAFIERDEGs, with detailed results summarized in Table 4. The analysis revealed that these genes were predominantly involved in biological processes (BP) such as regulation of response to transplantation in colorectal cancer (CRC), steroid catabolic process, and the regulation of axon and neuron projection regeneration. In terms of cellular components (CC), they were primarily associated with low-density lipoprotein particles, chylomicrons, endoplasmic reticulum lumen, late endosomes, and very-low-density lipoprotein particles. Regarding molecular functions (MF), the genes were mainly linked to cytokine activity, CXCR chemokine receptor binding, heparin binding, glycosaminoglycan binding, and sulfur compound binding. Additionally, significant enrichment was observed in the Toll-like receptor signaling pathway and pathways involving viral protein interactions with cytokines and cytokine receptors. The results were graphically represented using bar plots (Fig. 3A). Meanwhile, a network diagram was constructed to illustrate the associations among BP, CC, MF, and KEGG pathways based on the GO and KEGG enrichment results (Fig. 3B-E). In this network diagram, the connections represent the interactions between molecules; Each graphic element has detailed background information available for viewing. The size of a node intuitively reflects the number of molecules contained in that functional unit.

**Table 4:** Results of GO and KEGG enrichment analysis for CAFIERDEGs

ONTOLOGY	ID	Description	GeneRatio	BgRatio	pvalue	*p*.adjust	qvalue
BP	GO:1,903,034	regulation of response to wounding	3/7	173/18,870	2.58E-05	7.26E-03	2.54E-03
BP	GO:0031099	regeneration	3/7	191/18,870	3.47E-05	7.26E-03	2.54E-03
BP	GO:0006706	steroid catabolic process	2/7	25/18,870	3.52E-05	7.26E-03	2.54E-03
BP	GO:0048679	regulation of axon regeneration	2/7	30/18,870	5.11E-05	7.26E-03	2.54E-03
BP	GO:0070570	regulation of neuron projection regeneration	2/7	32/18,870	5.82E-05	7.26E-03	2.54E-03
CC	GO:0034362	low-density lipoprotein particle	1/7	12/19,886	4.22E-03	3.74E-02	1.92E-02
CC	GO:0042627	chylomicron	1/7	12/19,886	4.22E-03	3.74E-02	1.92E-02
CC	GO:0005788	endoplasmic reticulum lumen	2/7	313/19,886	4.92E-03	3.74E-02	1.92E-02
CC	GO:0005770	late endosome	2/7	315/19,886	4.98E-03	3.74E-02	1.92E-02
CC	GO:0034361	very-low-density lipoprotein particle	1/7	20/19,886	7.02E-03	3.74E-02	1.92E-02
MF	GO:0005125	cytokine activity	4/7	237/18,496	8.93E-07	3.21E-05	9.40E-06
MF	GO:0045236	CXCR chemokine receptor binding	2/7	18/18,496	1.87E-05	3.34E-04	9.77E-05
MF	GO:0008201	heparin binding	3/7	174/18,496	2.79E-05	3.34E-04	9.77E-05
MF	GO:0005539	glycosaminoglycan binding	3/7	240/18,496	7.27E-05	6.54E-04	1.91E-04
MF	GO:1,901,681	sulfur compound binding	3/7	275/18,496	1.09E-04	7.84E-04	2.29E-04
KEGG	hsa04620	Toll-like receptor signaling pathway	3/5	109/8875	1.77E-05	7.44E-04	5.40E-04
KEGG	hsa04061	Viral protein interaction with cytokine and cytokine receptor	2/5	100/8875	1.23E-03	2.58E-02	1.88E-02

### Construction of CRC subtypes

**Fig. 4:**
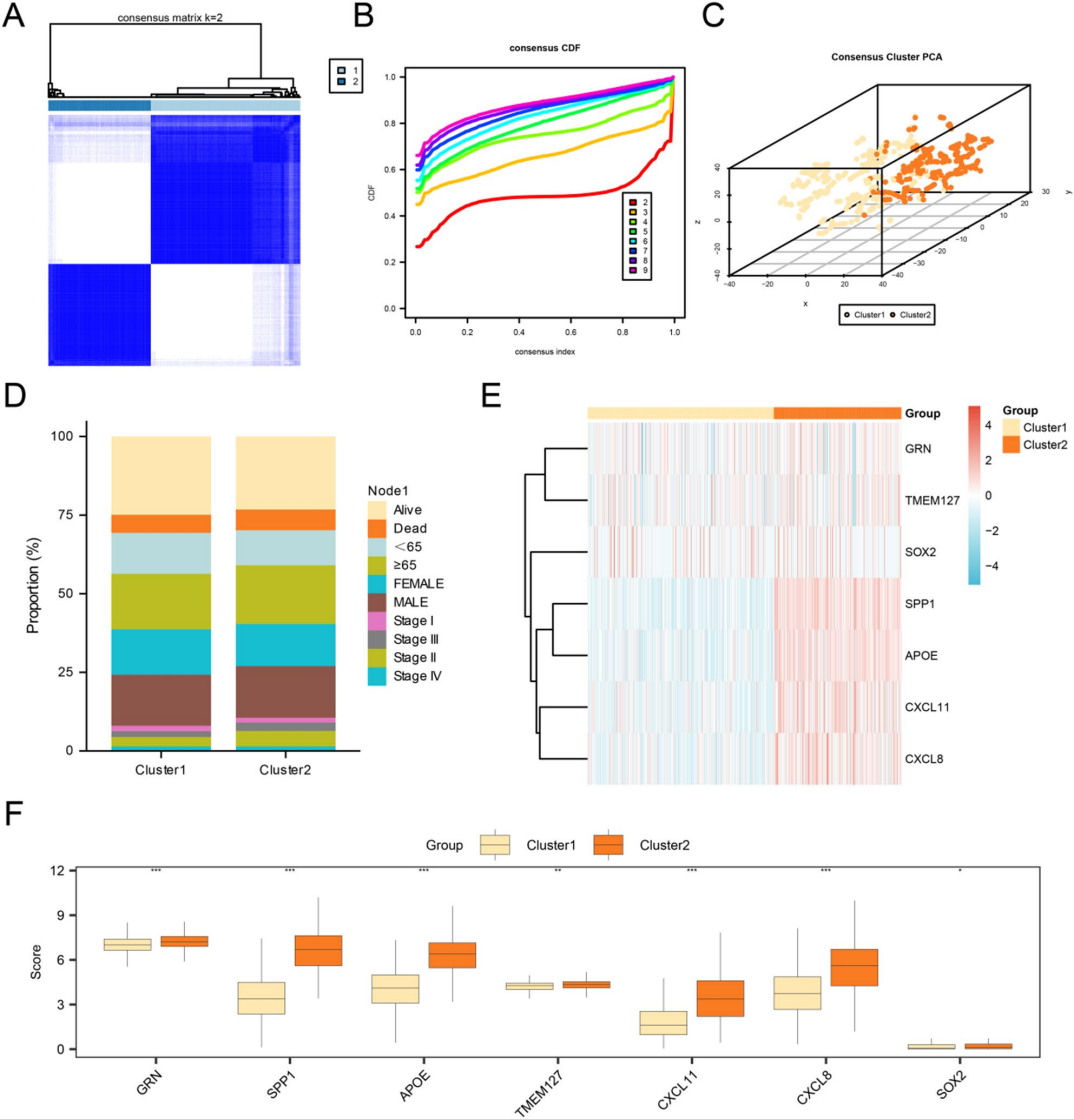
Consensus clustering analysis for CRC. **A** Plot of consensus clustering results for the CRC dataset (TCGA-COADREAD). **B** Consistency cumulative distribution function (CDF) of consistency clustering analysis. **C** 3D t-SNE cluster map of two disease subtypes of CRC. **D** Stacked bar plot of different clinical information in CRC subtypes. **E** Heat map of expression values in CRC subtypes. **F** Group comparison plot between two CRC subtype groups. * represents *p* < 0.05, statistically significant; ** represents *p* < 0.01, highly statistically significant; *** represents *p* value < 0.001, highly statistically significant

To explore the disease subtypes in the CRC dataset (TCGA-COADREAD), the R package ConsensusClusterPlus was used based on the expression levels of seven CAFIERDEGs. Consensus clustering analysis was employed to identify different disease subtypes of CRC samples, and two CRC subtypes were finally determined: subtype one (Cluster 1), containing 384 samples, and subtype two (Cluster 2), containing 263 samples (Fig. 4A-B). The results of the 3D t-SNE clustering map showed that the two disease subtypes were significantly different (Fig. 4C).

At the same time, combining the prognostic clinical information of CRC samples from the CRC dataset (TCGA-COADREAD), a stacked bar diagram of different clinical information among different disease subtypes was drawn (Fig. 4D). Subsequently, the R package pheatmap was employed to generate a heatmap illustrating the expression differences between the two CRC subtypes (Fig. 4E). Finally, to further validate the expression differences between CRC disease subtypes, the differential analysis results were presented based on the expression levels shown in the group comparison plot (Fig. 4F). The group comparison map indicated that the expression levels of seven CAFIERDEGs were statistically significant between the disease subtypes (*p* < 0.05). 

### Gene set enrichment analysis (GSEA) among CRC subtypes

**Fig. 5:**
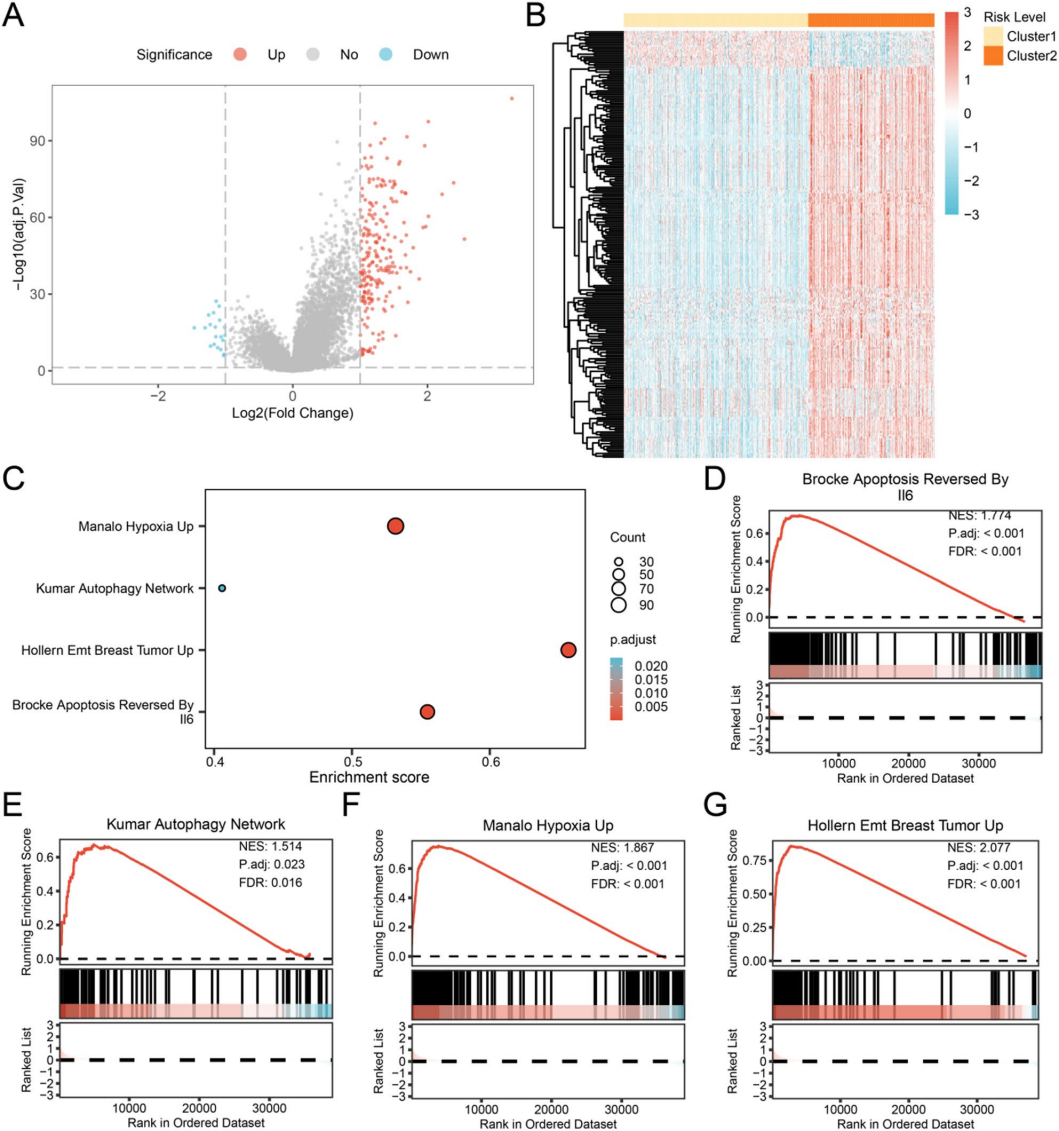
Differential gene expression analysis and GSEA for TCGA-COADREAD. **A**-**B** Volcano map (**A**) and heat map of expression values (**B**) of differentially expressed genes in Cluster 1 and Cluster 2 in the CRC dataset (TCGA-COADREAD). **C** Display of 4 biological function bubble plots of GSEA in the CRC dataset (TCGA-COADREAD). **D**-**G** GSEA showed that the CRC dataset (TCGA-COADREAD) was significantly enriched in BROCKE APOPTOSIS REVERSED BY IL6 (**D**), KUMAR AUTOPHAGY NETWORK (**E**), MANALO HYPOXIA UP (**F**), HOLLERN EMT BREAST TUMOR UP (**G**). In the bubble plot, the size of the bubble represents the size of the gene set, and the color of the bubble represents the size of the adj.p value. The more red the adj.p value, the smaller it is; the more blue the adj.p value, the larger it is. In the heatmap, red indicates high expression, while blue indicates low expression. The selection criteria for GSEA are adp < 0.05, FDR (q-value) < 0.25, and the p-value correction method is Benjamini-Hochberg (BH)

In order to further analyze the differences among CRC subtype one (Cluster 1) and CRC subtype two (Cluster 2) in the dataset (TCGA-COADREAD), the dataset was divided according to the consensus clustering results of the CRC model. The DEGs identified in the two groups were summarized as follows: A total of 239 DEGs in the TCGA-COADREAD dataset satisfied the criteria of |logFC| > 1 and adj.P.Val < 0.05. Among these, 219 genes were up-regulated (logFC > 1 and adj.P.Val < 0.05), whereas 20 genes were down-regulated (logFC < -1 and adj.P.Val < 0.05). Using the differential analysis results, we generated a volcano plot (Fig. 5A) to visualize the distribution of significantly up-regulated genes and a heatmap (Fig. 5B) to display the expression profiles of the DEGs.

To determine the effect of expression levels of all genes in the CRC dataset (TCGA-COADREAD) on the onset of CRC, based on the logFC values of all genes in the CRC dataset (TCGA-COADREAD) between Cluster 1 and Cluster 2, the relationship between the expression of all genes in the CRC dataset (TCGA-COADREAD) and biological processes, cellular components, and molecular functions was investigated by GSEA and presented as a bubble plot (Fig. 5C). The results were shown in Table 5. All genes in the CRC dataset (TCGA-COADREAD) were significantly enriched in BROCKE APOPTOSIS REVERSED BY IL6 (Fig. 5D), KUMAR AUTOPHAGY NETWORK (Fig. 5E), Brocke Apoptosis Reversed by IL6 (Fig. 5D), Kumar Autophagy Network (Fig. 5E), MANALO HYPOXIA UP (Fig. 5F), HOLLERN EMT BREAST TUMOR UP (Fig. 5G), and other biologically related functions and signaling pathways.

**Table 5:** Results of GSEA between cluster1 and cluster2 groups in TCGA-COADREAD

Description	setSize	enrichmentScore	NES	pvalue	*p*.adjust	qvalue
Hollern_emt_breast_tumor_up	140	0.859848668	2.077228766	1.00E-10	3.24E-09	2.23E-09
Manalo_hypoxia_up	205	0.754797778	1.867470708	1.00E-10	3.24E-09	2.23E-09
Brocke_apoptosis_reversed_by_il6	146	0.73089635	1.773542695	1.62E-07	2.77E-06	1.91E-06
Kumar_autophagy_network	69	0.674971378	1.514145437	4.97E-03	2.30E-02	1.59E-02

### Construction of prognostic risk model and clinical prognostic performance of CRC

**Fig. 6:**
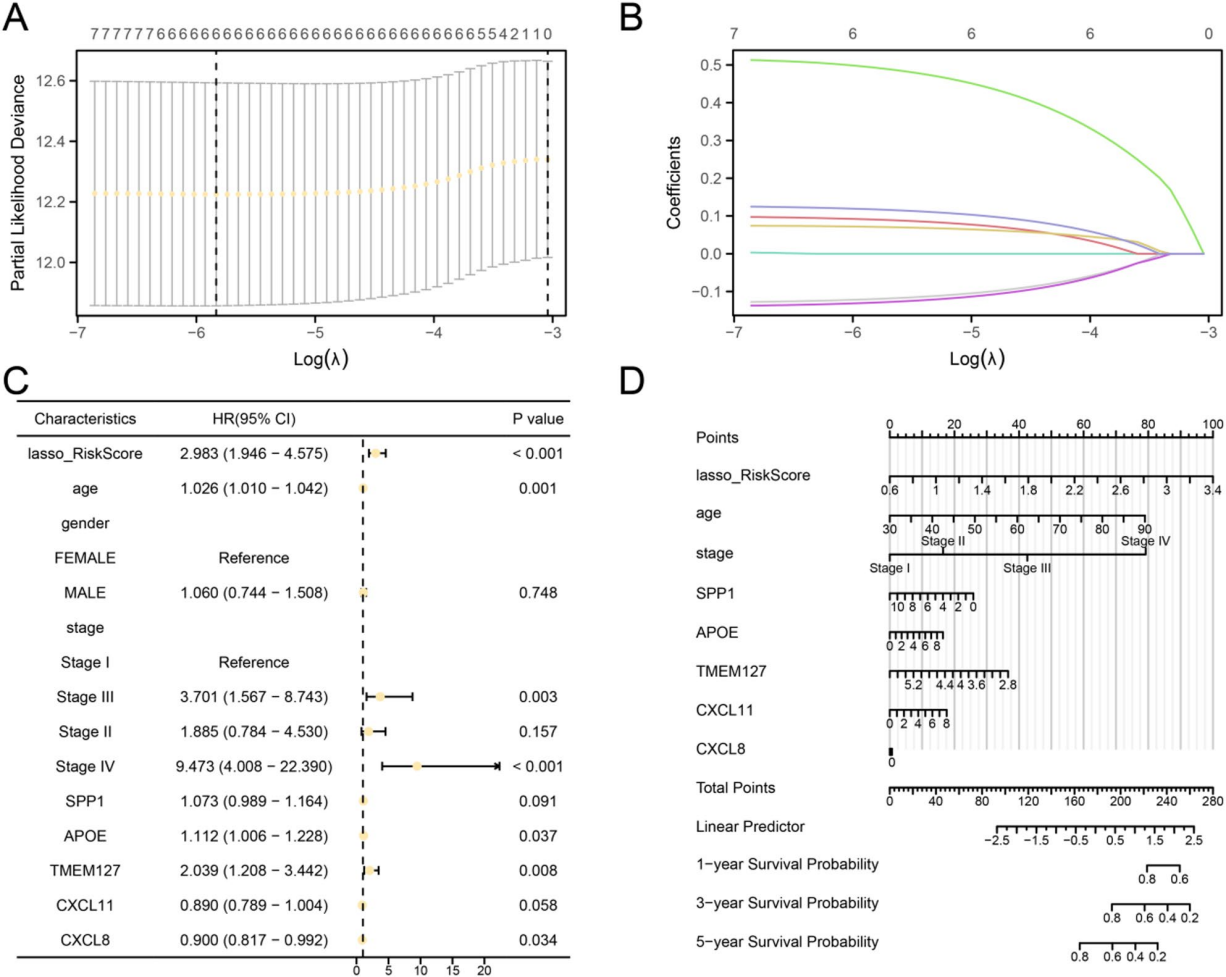
LASSO and cox regression analysis. **A**-**B**. Results of the LASSO Cox regression analysis.**A** the results obtained using 10-fold cross-validation to determine the optimal lambda value; the optimal parameter is lambda.min, corresponding to the minimum partial likelihood error. **B** the variation in gene regression coefficients across different lambda values. **C**-**D**. Forest plots (**C**) and scatter plots (**D**) of the LASSO risk score (RiskScore) and clinical information in the Cox regression model

To construct a prognostic risk model for colorectal cancer, a LASSO Cox regression analysis was performed on seven prognosis-related genes. Based on the results of 10-fold cross-validation, the lambda value corresponding to the minimum likelihood error (lambda.min) was selected as the optimal parameter. Under this condition, six key genes were identified: SPP1, APOE, TMEM127, CXCL11, CXCL8, and SOX2. The relevant results are shown in Fig. 6A-B.

$$\begin{aligned} \:\mathrm{R}\mathrm{i}\mathrm{s}\mathrm{k}Score\:&=\:\mathrm{S}\mathrm{P}\mathrm{P}1\mathrm{*}\left(0.091\right)+\mathrm{A}\mathrm{P}\mathrm{O}\mathrm{E}\mathrm{*}\left(0.071\right)\\&+\mathrm{T}\mathrm{M}\mathrm{E}\mathrm{M}127\mathrm{*}\left(0.493\right)+\mathrm{C}\mathrm{X}\mathrm{C}\mathrm{L}11\mathrm{*}(-0.120)\\&+\mathrm{C}\mathrm{X}\mathrm{C}\mathrm{L}8\mathrm{*}(-0.130)+\mathrm{S}\mathrm{O}\mathrm{X}2\mathrm{*}\left(0.118\right) \end{aligned}$$

Furthermore, based on the LASSO regression results, univariate and multivariate Cox regression models were conducted to investigate the expression levels of key genes, their association with clinical prognostic factors, and the predictive value of the LASSO-derived risk model for patient outcomes. Detailed information was presented in Table 6. The SOX2 gene was not included in this analysis because of collinearity or absence after removal following inclusion of multiple factors. Forest plots were used to illustrate the outcomes of the univariate Cox regression analysis (Fig. 6C). Variables with p values < 0.10 from the univariate analysis were incorporated into subsequent analyses. To further assess the value of the prognostic risk model for CRC, a nomogram was drawn based on the expression levels of Key Genes after screening; prognostic clinical features and LASSO risk models for multivariate Cox regression analysis were shown by this nomogram (Fig. 6D). The findings indicated that the LASSO risk score exhibited superior predictive performance compared to other variables in the CRC prognostic model. The contribution of key gene CXCL8 to the prognostic risk model for CRC is substantially lower compared to that of other variables.

**Table 6:** Cox regression analysis results in dataset TCGA-COADREAD

Characteristics	Total(*N*)	Univariate analysis		Multivariate analysis	
		Hazard ratio (95% CI)	*P* value	Hazard ratio (95% CI)	*P* value
lasso_RiskScore	622	2.983 (1.946–4.575)	< 0.001	2.847 (0.793–10.219)	0.109
Age	622	1.026 (1.010–1.042)	0.001	1.039 (1.022–1.057)	< 0.001
Gender	622				
Female	287	Reference			
MALE	335	1.060 (0.744–1.508)	0.748		
Stage	601				
Stage I	109	Reference		Reference	
Stage III	179	3.701 (1.567–8.743)	0.003	3.486 (1.454–8.357)	0.005
Stage II	226	1.885 (0.784–4.530)	0.157	1.625 (0.671–3.936)	0.282
Stage IV	87	9.473 (4.008–22.390)	< 0.001	10.198 (4.199–24.768)	< 0.001
SPP1	622	1.073 (0.989–1.164)	0.091	0.933 (0.779–1.119)	0.457
APOE	622	1.112 (1.006–1.228)	0.037	1.055 (0.879–1.267)	0.566
TMEM127	622	2.039 (1.208–3.442)	0.008	0.700 (0.302–1.620)	0.404
CXCL11	622	0.890 (0.789–1.004)	0.058	1.066 (0.862–1.319)	0.553
CXCL8	622	0.900 (0.817–0.992)	0.034	0.998 (0.821–1.213)	0.987

### Validation of prognostic risk models for CRC

**Fig. 7:**
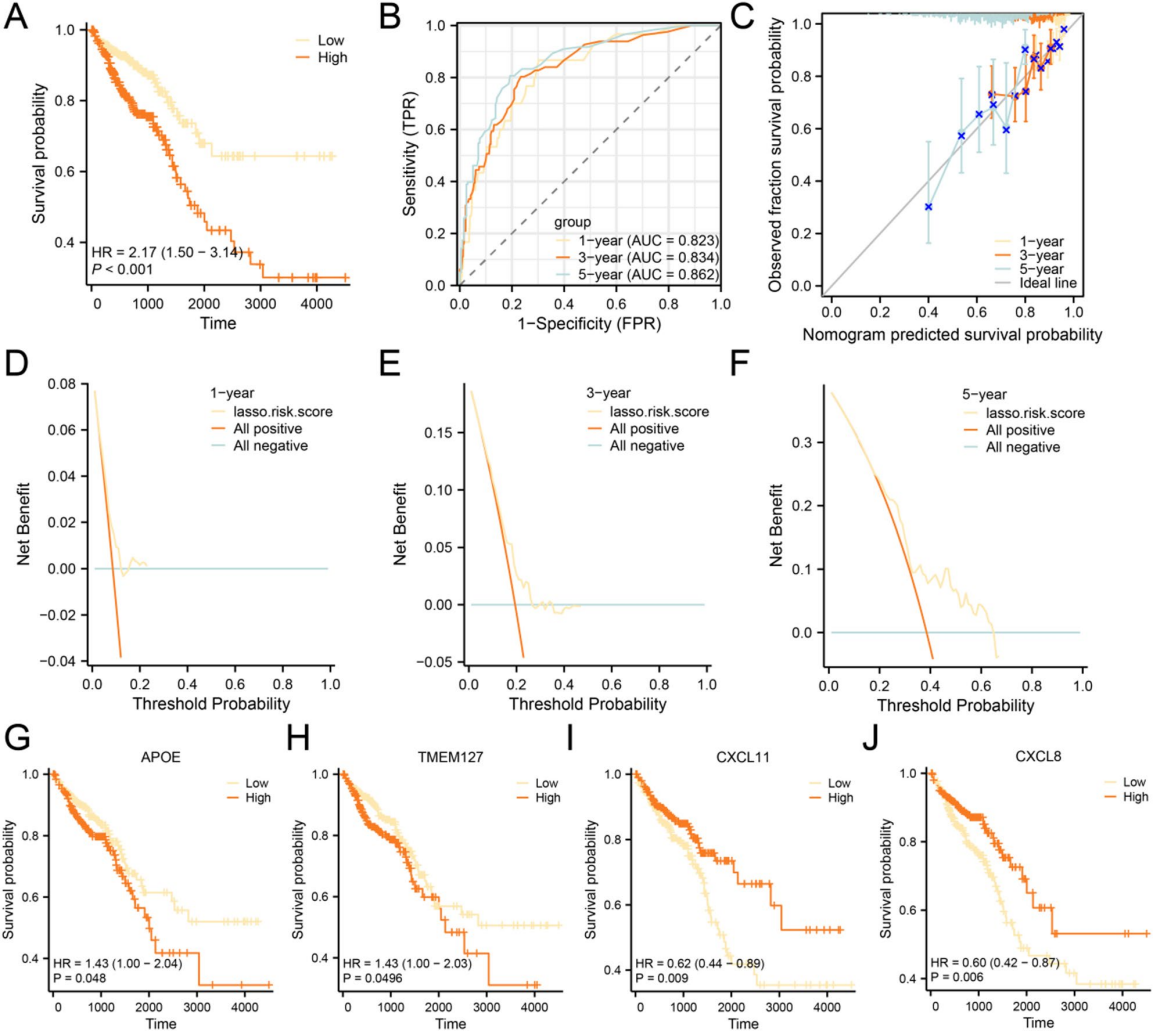
Prognostic analysis of TCGA-COADREAD. **A** Prognostic KM curves between high-risk and low-risk groups of LASSO risk score and OS of CRC in the CRC dataset (TCGA-COADREAD). **B** Time-dependent ROC curves of LASSO risk score in the CRC dataset (TCGA-COADREAD). **C** Calibration curves for the 1-, 3-, and 5-year prognostic risk model of CRC from the CRC dataset (TCGA-COADREAD). D-F 1 year (**D**), 3 years (**E**), and 5 years (**F**) DCA plots of the CRC prognostic risk model from the CRC dataset (TCGA-COADREAD). **G**-**J**. Prognostic KM curves between high-expression and low-expression groups of key genes APOE (**G**), TMEM127 (**H**), CXCL11 (**I**), and CXCL8 (**J**) combined with OS of CRC

Based on the LASSO risk score in the TCGA-COADREAD dataset, the KM survival curve was generated using median CRC sample OS stratification to evaluate prognostic outcomes (Fig. 7A). The analysis showed that there was a statistically significant difference in OS between the high-risk group and the low-risk group (*p* < 0.001). In addition, a time-dependent ROC curve was constructed for the same dataset (Fig. 7B), indicating that the prognostic risk model has certain predictive performance over time, with AUC values ranging from 0.7 to 0.9 during 1-, 3-, and 5-year follow-up periods.

Next, we conducted 1-, 3-, and 5-year prognostic calibration assessments using the CRC dataset (TCGA-COADREAD) to evaluate the predictive accuracy of the risk model, and generated corresponding calibration plots (Fig. 7C). In these plots, the horizontal axis indicates the model-predicted survival probabilities. The vertical axis of the calibration curves reflects the actual observed survival probabilities, where model-predicted lines that are closer to the ideal reference (gray line) at various time points suggest higher prediction accuracy. The findings demonstrate that the prognostic risk model for CRC achieves optimal clinical predictive capability at the 5-year follow-up point. Concurrently, decision curve analysis (DCA) was employed to assess and illustrate the clinical applicability of the CRC prognostic risk model at 1-year (Fig. 7D), 3-year (Fig. 7E), and 5-year (Fig. 7F) intervals. The findings revealed that the multivariable Cox regression models developed in this study exhibited the highest clinical predictive value at the 5-year time point, followed by the 3-year and then the 1-year predictions.

Subsequently, we conducted KM curve analyses (Fig. 7G-J) using median-based stratification of OS in CRC, integrating key gene expression profiles with the TCGA-COADREAD dataset. The analysis revealed that four key genes: APOE, TMEM127, CXCL11, and CXCL8 showed statistically significant differences (*p* < 0.05) in OS between high-risk and low-risk groups within the CRC cohort.

**Fig. 8:**
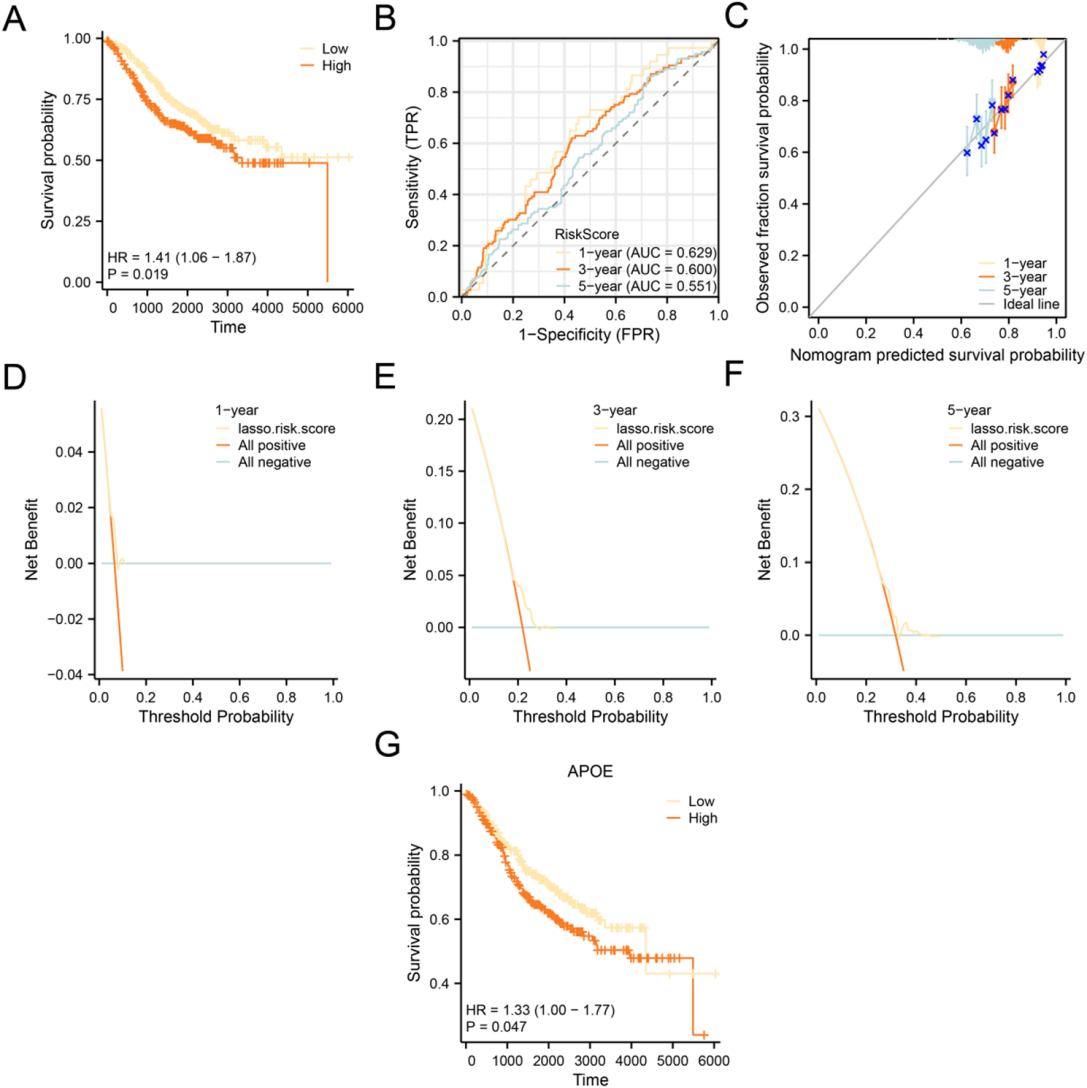
Prognostic analysis of GSE39582. **A** Prognostic KM curves between high-risk and low-risk groups of LASSO risk score and OS of CRC in the CRC dataset (GSE39582). **B** Time-dependent ROC curves for LASSO risk score in the CRC dataset (GSE39582). **C** Calibration curves for the 1-, 3-, and 5-year prognostic risk model from the CRC dataset (GSE39582). D-F. DCA plots for 1 year (**D**), 3 years (**E**), and 5 years (**F**) of the CRC prognostic risk model from the CRC dataset (GSE39582). **G** Prognostic KM curves between high-expression and low-expression groups of Key Genes APOE combined with OS of CRC

In addition, we performed prognostic KM curve analyses (Fig. 8A) based on median grouping for OS of CRC samples in the LASSO risk score combined with the CRC dataset (GSE39582). The results indicated that there was a statistically significant difference in OS between the high-risk group and the low-risk group regarding the CRC samples in the CRC dataset (GSE39582) (p value < 0.05). In addition, time-dependent ROC curves (Fig. 8B) were plotted for the CRC samples in the CRC dataset (GSE39582). The results indicated that the prognostic risk model for CRC exhibited low accuracy over 1 year, 3 years, and 5 years (0.7 > AUC > 0.5).

We conducted 1-, 3-, and 5-year prognostic calibration assessments using the CRC dataset (GSE39582) to evaluate the predictive accuracy of the risk model, and generated corresponding calibration plots (Fig. 8C). The results indicated that the CRC prognostic risk model exhibits the best clinical predictive performance at the 5-year mark. Meanwhile, DCA was applied to assess and visualize the clinical usefulness of the CRC prognostic risk model at 1 year (Fig. 8D), 3 year (Fig. 8E), and 5 year (Fig. 8F) time points. The results demonstrated that the multivariable Cox regression models developed in this study provided the highest clinical predictive benefit at the 5-year mark, followed by the 3 year and 1 year intervals, respectively. Finally, we conducted KM survival analyses using median-based stratification derived from key gene expression levels in conjunction with OS data of CRC patients from the GSE39582 dataset (Fig. 8G). The analysis revealed that the key gene APOE exhibited a statistically significant difference in OS between high-risk and low-risk groups (*p* < 0.05) within this cohort.

### Protein-protein interaction (PPI) network and hub gene screening

**Fig. 9:**
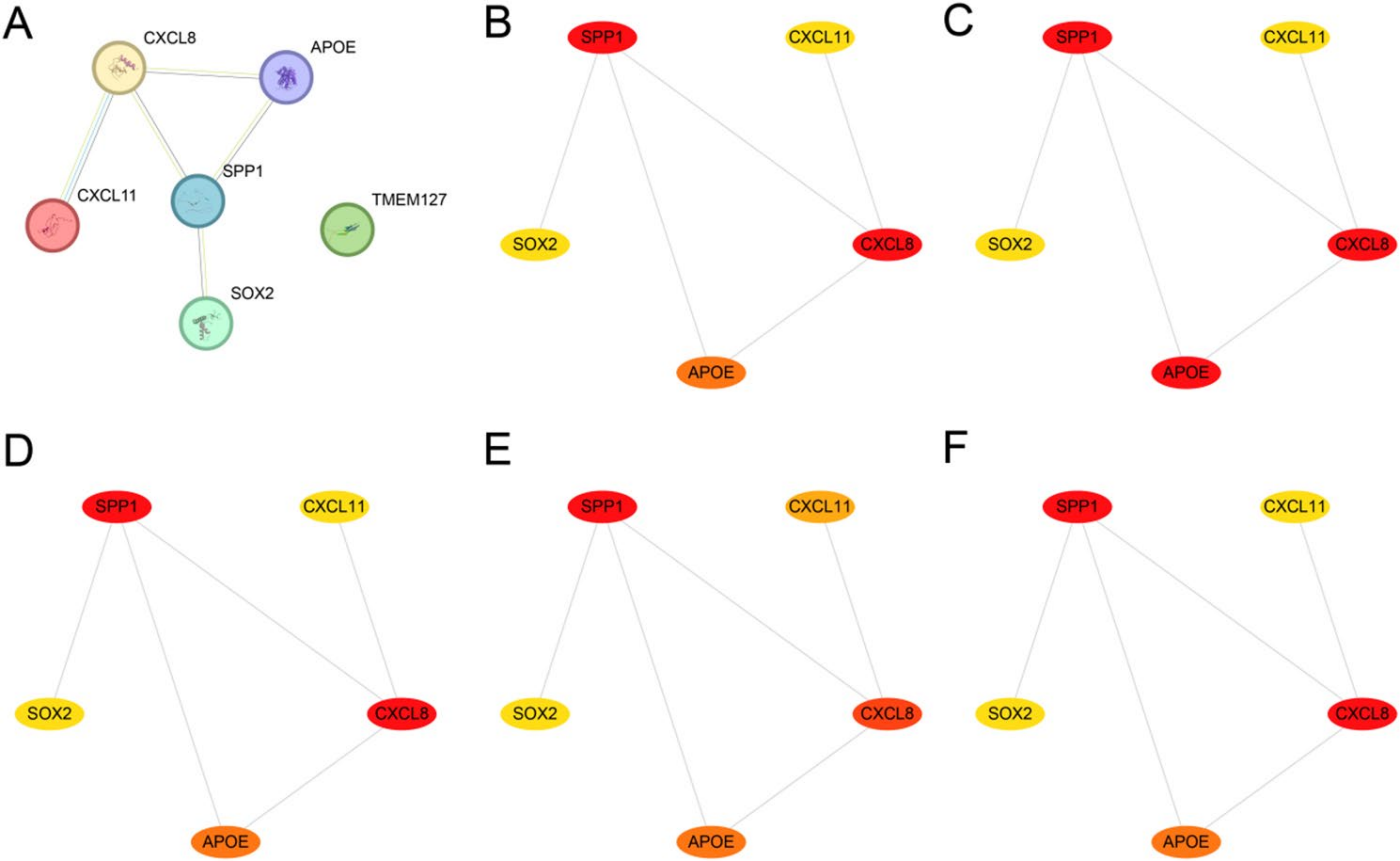
PPI network and hub genes analysis. **A** Utilize the STRING database to calculate the PPI network of key genes. **B**-**F** The PPI network of hub genes is calculated using five algorithms from the CytoHubba plugin, including MCC (**B**), MNC (**C**), Degree (**D**), EPC (**E**), and Closeness **F**

First, a PPI analysis was performed using the STRING database to construct a PPI network encompassing the six key genes (Fig. 9A). The PPI network results indicated that five key genes were associated, namely SPP1, APOE, CXCL11, CXCL8, and SOX2. These five key genes were defined as hub genes associated with CRC. Subsequently, the scores of the five hub genes were calculated using the CytoHubba plugin of Cytoscape software, employing five different algorithms. The hub genes were prioritized according to scores derived from five different algorithms: Maximum Clique Centrality (MCC), Degree, Maximum Neighborhood Component (MNC), Edge Percolated Component (EPC), and Closeness centrality. Then, the PPI networks were drawn for the hub genes according to the five algorithms: MCC (Fig. 9B), MNC (Fig. 9C), Degree (Fig. 9D), EPC (Fig. 9E), and Closeness (Fig. 9F). Among them, the color of the circles from red to yellow represents the score from high to low.

### Immune infiltration analysis between high and low risk groups (CIBERSORT)

**Fig. 10:**
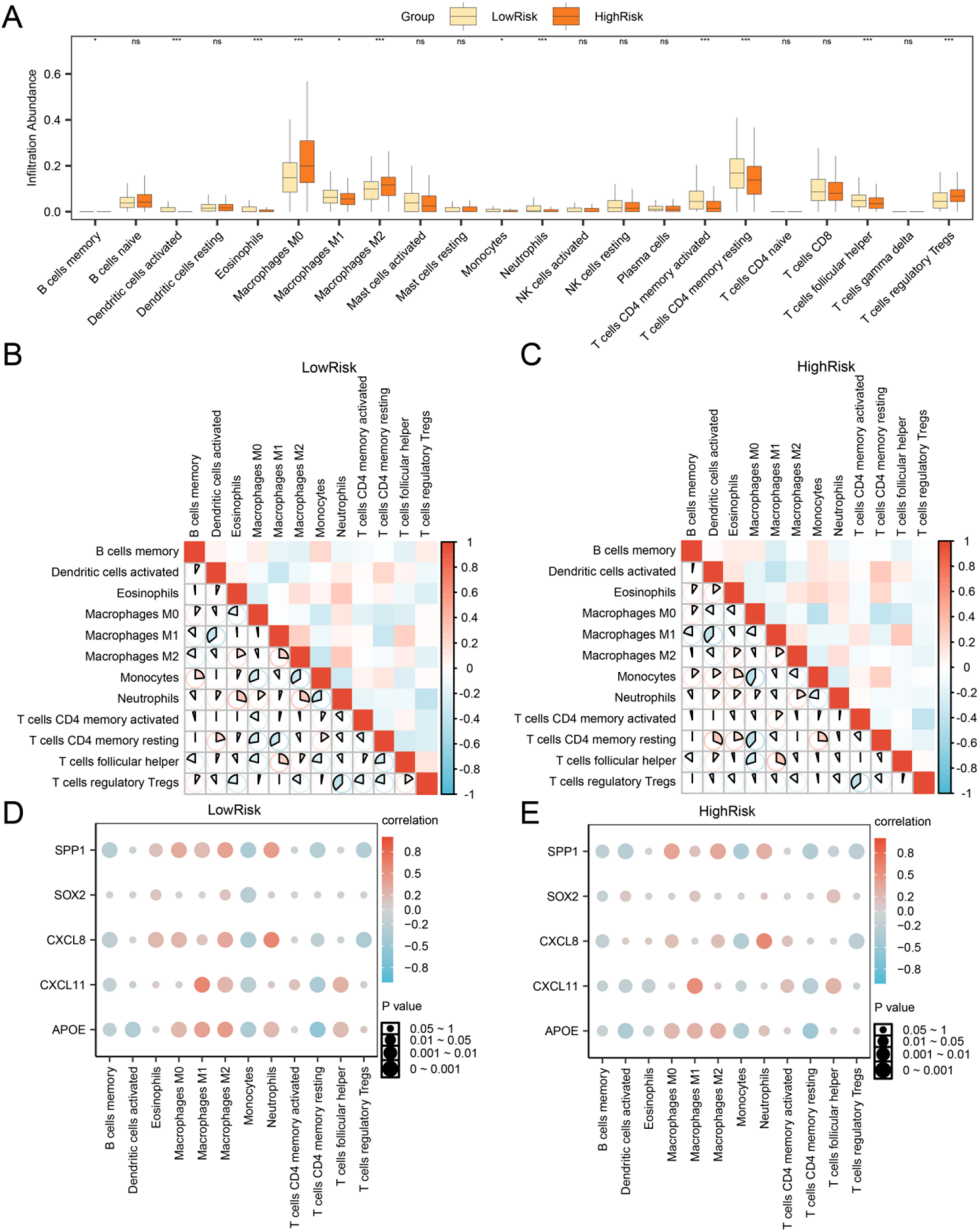
Immune infiltration analysis by CIBERSORT algorithm. **A** Plot of grouping comparison of immune cells in the low-risk and high-risk groups of CRC samples. B-C. Correlation heat map of immune cells in the low-risk (**B**) and high-risk (**C**) groups of CRC samples. D-E. Bubble plot of correlation between immune cell infiltration abundance and hub genes in the low-risk (**D**) and high-risk (**E**) groups of CRC samples.ns stands for *p* ≥ 0.05, not statistically significant; * represents *p* < 0.05, statistically significant; ** represents *p* < 0.01, highly statistically significant; *** represents *p* < 0.001, highly statistically significant. The absolute value of correlation coefficient (r value): below 0.3 was weak or no correlation, between 0.3 and 0.5 was weak correlation, between 0.5 and 0.8 was moderate correlation, and above 0.8 was strong correlation. Red indicates a positive correlation, while blue indicates a negative correlation. The depth of the color represents the strength of the correlation

The CIBERSORT algorithm was used to calculate the immune infiltration abundance of 22 immune cells in the CRC dataset (TCGA-COADREAD). First, the inter-group comparison plot illustrates variations in immune cell infiltration levels among different groups based on the outcomes of the immune infiltration analysis. The intergroup comparison plot (Fig. 10A) displays the distribution of 12 immune cell types: memory B cells, resting and activated CD4 + memory T cells, follicular helper T cells, regulatory T cells (Tregs), monocytes, M0, M1, and M2 macrophages, activated dendritic cells, eosinophils, and neutrophils; all exhibiting statistically significant differences (*p* < 0.05).

Subsequently, a correlation heatmap was generated to illustrate the associations among the abundances of 12 immune cell types infiltrating CRC samples (Fig. 10B-C). The analysis revealed that immune cells in the low-risk group generally displayed strong inter-correlations, with Tregs and neutrophils showing the most pronounced negative correlation (*r* = -0.374, *P* < 0.05) (Fig. 10B). Most immune cells in the high-risk group also showed strong correlations, with monocytes and M0 macrophages exhibiting the most pronounced significant negative correlation (*r* = -0.422, *p* < 0.05) (Fig. 10C). Finally, the association between hub genes and levels of immune cell infiltration was depicted through bubble plots (Fig. 10D–E). The correlation analysis revealed a strong relationship between most immune cells and hub genes in the low-risk group. CXCL11 exhibited the strongest positive correlation with M1 macrophages (*r* = 0.607, *P* < 0.05) (Fig. 10D); furthermore, a significant positive association was observed between CXCL8 and neutrophils (*r* = 0.583, *p* < 0.05) in the high-risk group (Fig. 10E).

### Immunotherapy analysis of hub genes

**Fig. 11:**
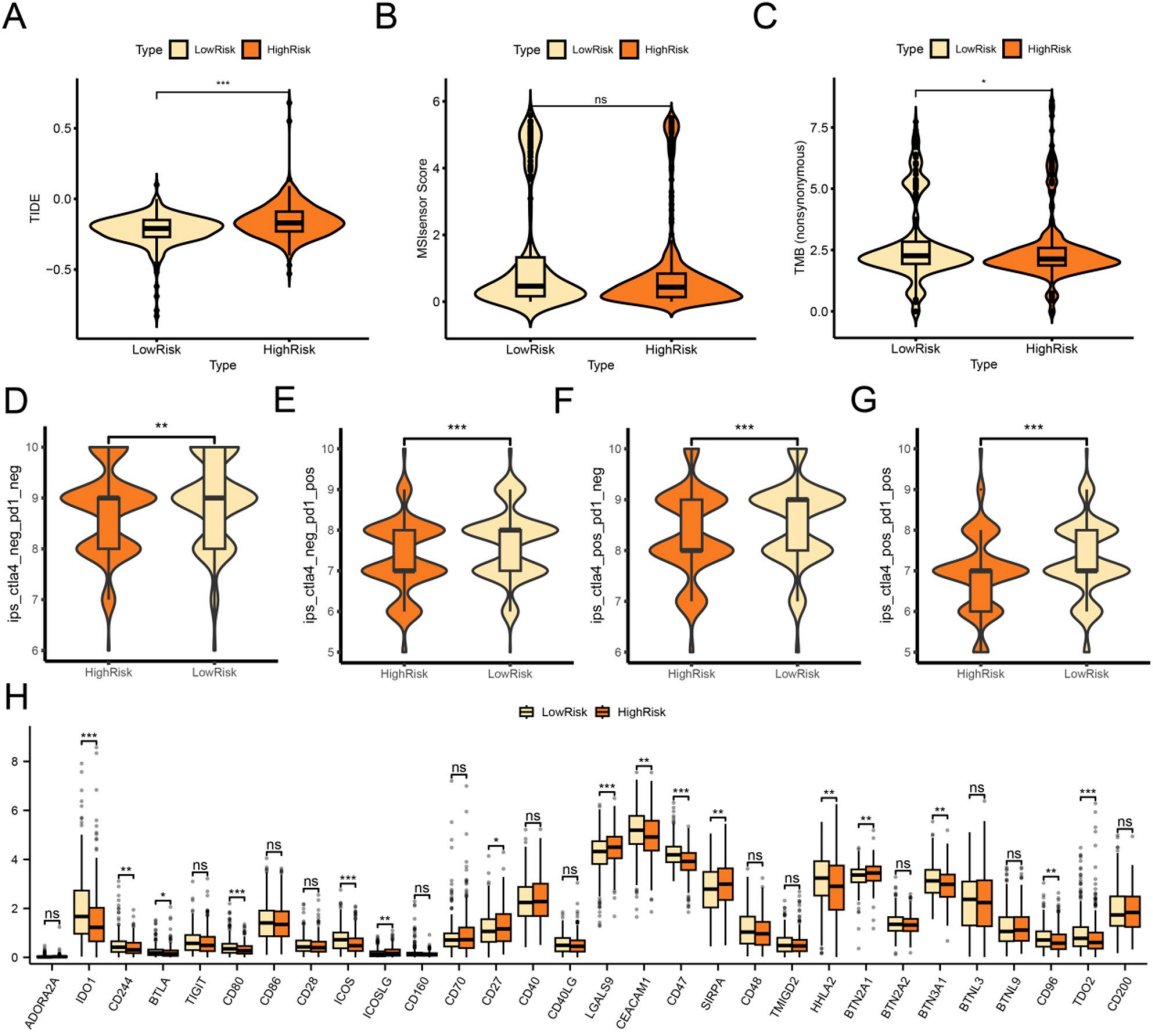
Immunotherapy analysis of hub gene. Results for TIDE immunotherapy score (**A**),MSI (**B**), TMB score (**C**) ,group comparison of results for CRC between high-risk and low-risk groups in the CRC dataset (TCGA-COADREAD) were shown. D-G.Immunogenicity score (IPS) ips_ctla4_neg_pd1_neg (**D**), ips_ctla4_neg_pd1_pos (**E**), ips_ctla4_pos_pd1_neg (**F**), ips_ctla4_pos_pd1_neg (**F**), ips_ctla4_neg_pd1_pos (**E**). Comparison of ips_ctla4_pos_pd1_pos (**G**) grouping between high-risk and low-risk groups of CRC in the CRC dataset (TCGA-COADREAD). **H** Immune checkpoint groups comparison diagram of CRC between high-risk and low-risk groups in the CRC dataset (TCGA-COADREAD). ns stands for *p* ≥ 0.05, not statistically significant; * represents *p* < 0.05, statistically significant; ** represents *p* < 0.01, highly statistically significant; *** represents *p* < 0.001, highly statistically significant

This study analyzed the TCGA-COADREAD dataset using the TIDE algorithm to evaluate the immunotherapy sensitivity of CRC, and the results were presented in the comparison group figure (Fig. 11A). The results showed that the TIDE immunotherapy score was highly statistically significant among different subtypes (*p* < 0.001), and that the low-risk group had a lower score than the high-risk group. Therefore, it is reasonable to conclude that the therapeutic efficacy of immunotherapy is potentially superior in the low-risk population. We then analyzed the differences in MSI and TMB scores between high-risk and low-risk CRC groups. The comparison between MSI score groups (Fig. 11B) showed that there was no statistically significant difference between the high-risk group and the low-risk group (*p* > 0.05). However, the comparison between TMB score groups indicated that there was a statistically significant difference between these two groups (*p* < 0.05), with the TMB score of the high-risk group being significantly lower than that of the low-risk group (Fig. 11C).

Analysis of the Immunogenicity Score (IPS) using data from the TCIA database revealed a significant difference in IPS between the high-risk and low-risk groups of CRC (TCGA-COADREAD) (*p* < 0.05) (Fig. 11D-G), with the high-risk group showing lower predicted efficacy for immunotherapy. Finally, we obtained a total of 30 immune checkpoint genes in the CRC dataset (TCGA-COADREAD) and mapped their expression profiles between the high-risk and low-risk groups (Fig. 11H). The results showed that most immune checkpoint genes were significantly highly expressed in the low-risk group.

### The expression of hub genes on CRC-related CAFs in mice was verified by qPCR

**Fig. 12:**
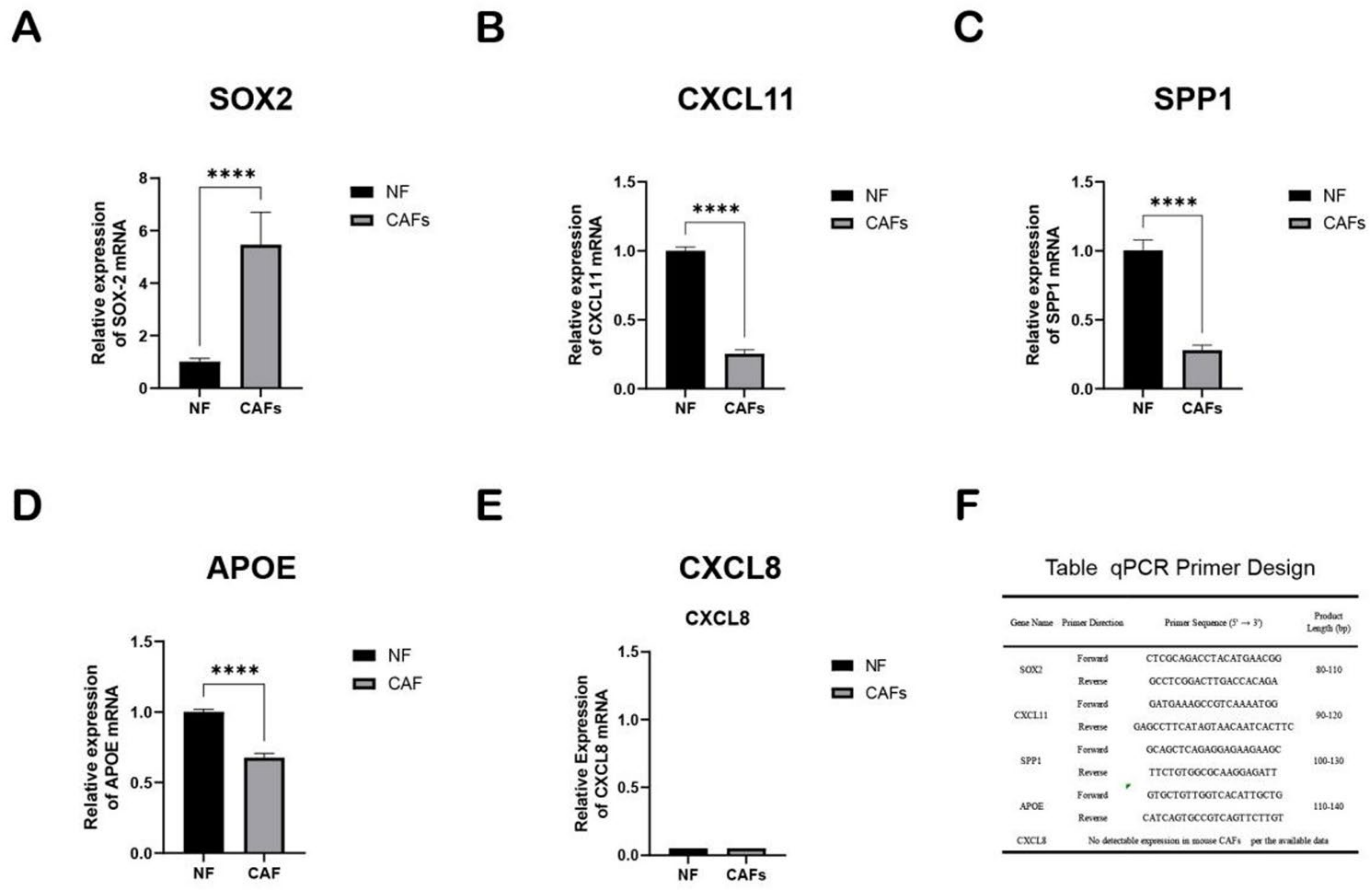
Validation of hub gene expression and qPCR primer design. **A**-**E** Relative mRNA expression levels of the identified hub genes SOX2, CXCL11, SPP1, APOE, and CXCL8 were determined by quantitative qPCR and are presented as mean ± SD from three independent experiments. Data were presented as the mean ± SD from three independent experiments. Statistical significance was determined by a Student’s t-test (****p < 0.001). Notably, we have not yet found any CXCL8 primers for mice. **F** Table listing the primer sequences used for qPCR amplification of the corresponding genes, including primer direction, sequence (5′ → 3′), and expected product length

Both CXCL11 and SPP1 exhibited low expression levels in CAFs isolated from mouse CRC tissues. In contrast, SOX2 showed high expression in these cells, a finding that contrasts with the results predictions of database filtering. This discrepancy suggested that the regulatory mechanisms or functional roles of these three genes may be influenced by species-specific factors and the complexity of the TME. Additionally, APOE was found to be downregulated in CAFs from mouse CRC tissues, aligning with database filtering results. We have not yet found any CXCL8 primers for mice. (Fig. 12).

### Immunohistochemical expression of hub genes

**Fig. 13:**
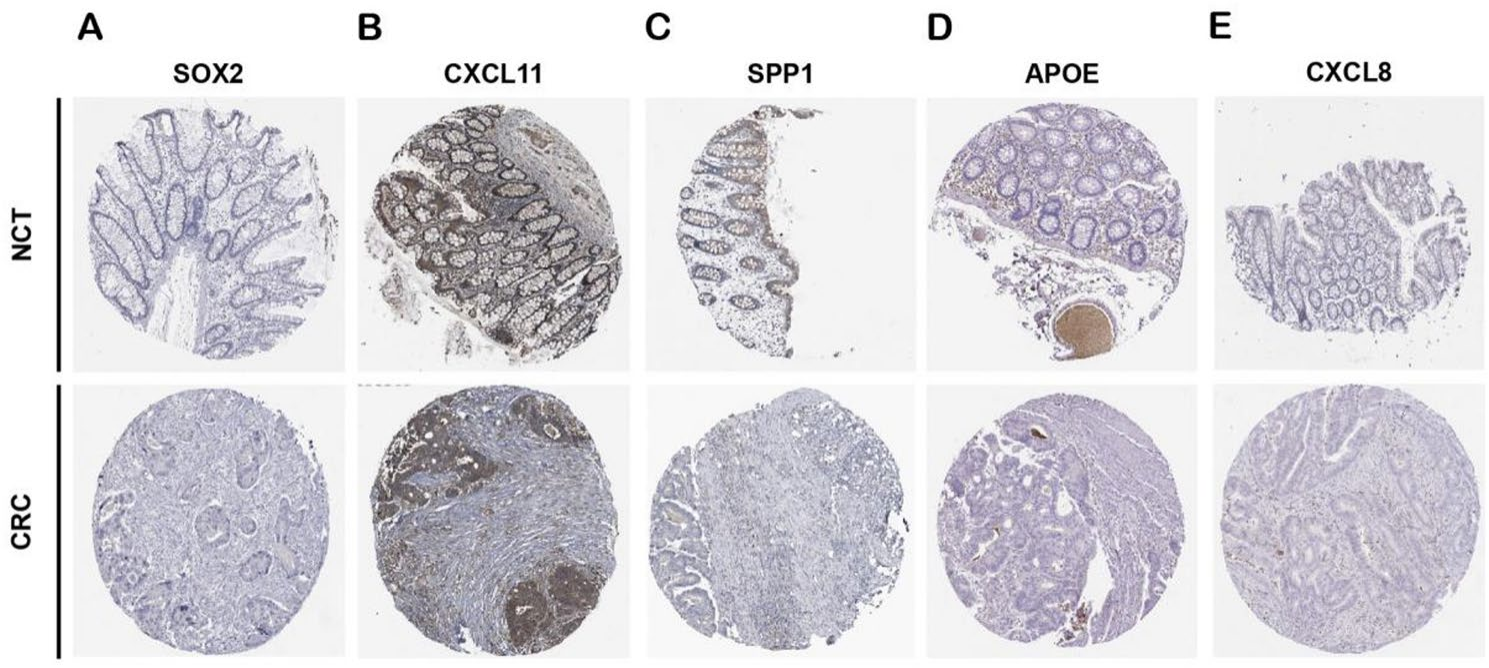
Immunohistochemical validation the expression of hub gene proteins. Immunohistochemical staining of the five identified hub genes in paired normal colon tissues (upper row) and CRC tissues (lower row). (**A**) SOX2, (**B**) CXCL11, (**C**) SPP1, (**D**) APOE, and (**E**) CXCL8. Representative images from circular tissue sections demonstrate protein localization patterns in the tissue microenvironment

The immunohistochemical analysis based on The HPA database demonstrated that, compared to normal intestinal mucosa, CXCL8, CXCL11, and SPP1 exhibited high expression levels in the human CRC tumor stroma, whereas APOE and SOX2 showed low expression in the tumor stroma. These results were consistent with those of the database filtering. (Fig. 13).

### Expression profiling of hub genes in single-cell sequencing of CRC FFPE samples

**Fig. 14:**
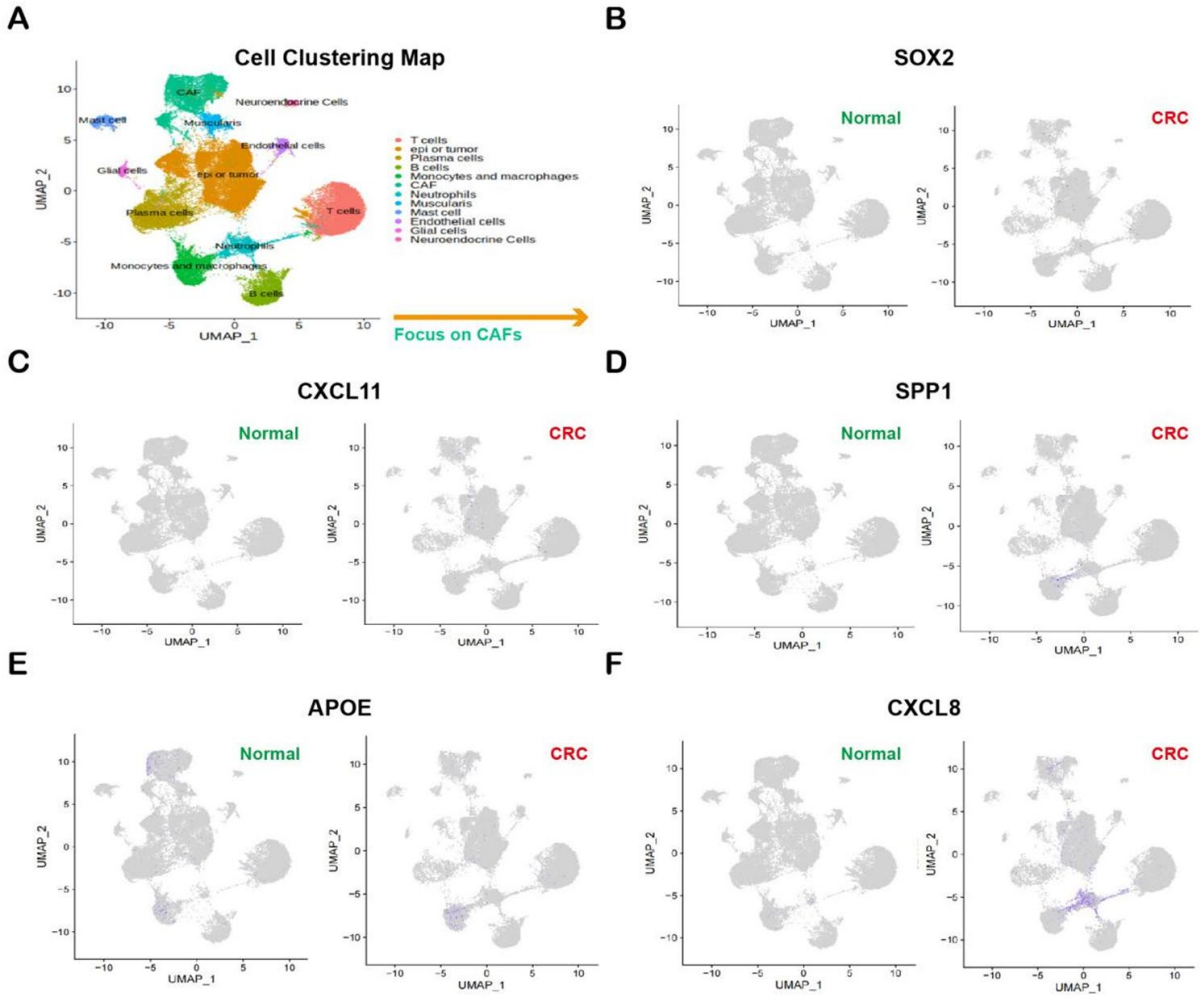
Single-cell analysis reveals the expression profiles of hub genes in CAFs. (**A**) A cell clustering map of a CRC sample generated from single-cell RNA sequencing highlights the CAF population (indicated by the arrow). (**B**-**F)** Dot plots showing the expression levels of the following hub genes in paired normal (left) and CRC (right) tissues at the single-cell level: (**B)** SOX2, (**C**) CXCL11, (**D**) SPP1, (**E**) APOE, and (**F**) CXCL8

Through single-cell sequencing of FFPE samples obtained from five cases of CRC and their corresponding adjacent normal tissues, we constructed the single-cell landscape of CRC, which encompasses twelve major cell types, including T cells, B cells, and CAFs.In this landscape, we found that CAFs highly expressed CXCL8, CXCL11, and SPP1, whereas APOE exhibited low expression in these cells compared to fibroblasts in normal tissues(Fig. 14).

## Discussion

One reason for the high mortality rate of CRC is its invasiveness and tendency to metastasize, often spreading to the liver, lungs, and other organs, which affects survival rates. Some patients have molecular biological characteristics, such as *KRAS* and *BRAF* gene mutations or low microsatellite instability, that lead to poor responses to chemotherapy and targeted therapy. Searching for potential biomarkers is significant for early CRC diagnosis and adjuvant treatment. Tumor immune escape is the phenomenon in which tumor cells evade recognition and attack by the immune system through various mechanisms, allowing them to survive, proliferate, and develop within the body. Tumor cells evade immune clearance by upregulating immune checkpoint molecules and reshaping the immunosuppressive microenvironment. This directly affects the response rate and persistence of immunotherapy [44].CAFs are an important component of TME. CAFs and other cellular components secrete immunosuppressive factors, such as TGF-β, IL-10 and VEGF. These molecules inhibit the activity of effector T cells while promoting the expansion of regulatory T cells and myeloid-derived suppressor cells. This contributes to the development of an immunosuppressive microenvironment [45].Immune escape is now considered a potential prognostic indicator and therapeutic target for cancer patients.This study aims to comprehensively analyze CAFs and tumor immune escape and explore their roles and potential mechanisms in CRC. A comparative assessment of gene expression profiles in CRC and normal tissues was performed using data from a public database. Through this analysis, 34 CAFIERDEGs were identified that exhibited significant expression differences between the two groups. Seven genes were found to be upregulated in CRC samples, while 27 others showed higher expression levels in the normal control group. Univariate Cox regression analysis ultimately yielded seven genes related to prognosis: GRN, SPP1, APOE, TMEM127, CXCL11, CXCL8, and SOX2. Enrichment analysis of GO and KEGG revealed that the seven genes play multiple roles in CRC pathogenesis, including regulating immune responses, influencing metabolic processes, and participating in cellular signaling pathway interactions. These results provide an important foundation for subsequent research to further explore the biological characteristics of CRC and potential treatment strategies. We developed and validated a LASSO regression model that incorporates five critical genes—SOX2, SPP1, APOE, CXCL11, and CXCL8—which effectively distinguishes between high- and low-risk patient groups. This model demonstrates optimal predictive performance at the 5-year clinical endpoint. This prognostic risk model serves as a novel tool for the clinical evaluation of patient outcomes. Additionally, the PPI network revealed interconnections among the five hub genes. The network highlights the intricate functional relationships between these key proteins and offers insights into their biological roles in CRC. This lays the groundwork for future experimental validation. Finally, analyses of immune infiltration and immunotherapy response were conducted in high- and low-risk CRC patient cohorts. This approach can improve treatment efficacy and the accuracy of outcome predictions, reveal potential biological differences between risk groups, and provide a solid foundation for optimizing future treatment regimens.

Then, through qPCR experiments, we verified the mRNA expression of CXCL11, SPP1, and SOX2 in mouse CRC CAFs and found that it was contrary to the results of database screening. One possible reason was that species-specific differences cannot be ignored. Although the human and mouse genomes are highly homologous, the transcriptional regulatory elements and splicing variants of certain matrix-associated genes exhibit species-specific characteristics. These characteristics may result in differences in basal expression levels and activation-induced patterns in mouse fibroblasts compared to human fibroblasts. Second, the heterogeneity of CAFs dictates dynamic changes in their gene expression profiles, particularly in activated subpopulations. Specific human CAF subpopulations identified through database analysis may be underrepresented or insufficiently induced in mouse models [46]. Furthermore, the persistent presence of TME signals is crucial. CAFs in human tumors undergo long-term chronic stimulation by tumor cells, and their high gene expression state depends on continuous paracrine signals and matrix interactions [47]. In contrast, mouse CAFs in isolated cultures or short-term tumor xenograft models lack continuous tumor stimulation and are detached from the real TME. Their activated state may rapidly decline, leading to differences in target gene expression.Then, we found in the HPA database that the protein expression of the five aforementioned genes in the immunohistochemistry of human CRC tissues was consistent with the database’s screening results. Finally, we performed single-cell sequencing on FFPE samples from 5 cases of human CRC and found that the results were consistent with those of the database screening.

Within the TME, molecules including SOX2,SPP1, APOE, CXCL11, and CXCL8 play a critical role in mediating communication between tumor cells, CAFs, and TAMs. This facilitates immune escape and advances tumor development. SPP1 can be expressed by tumor cells, TAMs, and certain CAF subsets. Spatial transcriptome and single-cell analyses revealed intercellular interactions between FAP^+^ fibroblasts and SPP1^+^ macrophages, which helped the ECM reshape and coordinate the formation of a pro-fibrotic microenvironment. This microenvironment prevented lymphocyte infiltration into the tumor core and reduced the efficacy of PD-L1 treatment [48].SPP1 can directly bind to cell surface receptors, including ITGB1 and CD44. This binding promotes the transformation of fibroblasts into an activated phenotype. This transformation influences the functional state and matrix remodeling of immune cells [49]. Activating CAF can secrete TGF-β, CXCL12 and other factors. These factors can induce tumor or myeloid cells to upregulate SPP1, creating a positive feedback loop that amplifies the local effect.This type of amplification loop can explain the co-enrichment of SPP1^+^ regions and highly fibrotic or immunosuppressive regions observed in some tumor samples. It also makes it difficult for a single targeting strategy to completely reverse the immunosuppressive state. SPP1 promotes the secretion of CXCL12 by activating the β-catenin/HIF-1α pathway in CAFs. SPP1 induces epithelial-mesenchymal transition in tumor cells to promote metastasis and inhibits the infiltration of CD8^+^ T cells to form an immunosuppressive TME, ultimately leading to tumor cell escape and immunotherapy resistance [50].This dual-layered immunosuppressive environment explains why targeting the PD-1/PD-L1 pathway alone has limited success in tumors with elevated SPP1 levels or extensive CAFs activation. Thus, tumor immune escape is not limited to the expression of checkpoint molecules, but rather involves the coordinated suppression of various stages, including cellular entry, the recruitment of immune populations, and metabolic interference [51].

ApoE is a protein related to lipid metabolism that also plays a role in modulating the TME. It is produced and released by tumor cells, TAMs, and certain stromal components, including CAFs. ApoE secreted by tumor cells activates the SYK-ERK pathway by binding to TREM2 on the surface of neutrophils, inducing senescence and inhibiting apoptosis. Senescent neutrophils SASP, including IL-6, which enhances immunosuppressive and tumor-promoting activities [52]. In models of pancreatic neuroendocrine tumors, mTOR inhibitors have been shown to decrease ApoE secretion by tumor cells, leading to the suppression of extracellular matrix remodeling. This indicates that ApoE-mediated activation of CAFs is regulated by the mTOR signaling pathway. These results suggest a promising therapeutic approach involving the combination of matrix-directed treatments, such as pairing mTOR inhibition with ECM-targeting strategies [53]. CXCL11, a chemokine that binds to the CXCR3 receptor, recruits T cells and NK cells, thereby enhancing immune surveillance and attacks on tumor cells [54]. Compared to normal fibroblasts, CAFs secrete significantly higher levels of CXCL11, which promotes tumor cell migration and metastatic progression. In hepatocellular carcinoma, CAFs produced CXCL11 increases tumor cell invasiveness by regulating the circUBAP2/miR-4756/IFIT1/3 signaling cascade [55]. CXCL11 enhances the migration and activity of CD8^+^ T cells by binding to the CXCR3 receptor and forming an anti-tumor immune response. These findings suggest the potential to promote anti-tumor effects through the CXCL11/CXCR3 axis, opening up a new direction for cancer treatment research [56]. CXCL8, also known as IL-8, is produced by tumor cells and tumor-associated stromal cells. It is a potent neutrophil chemokine that promotes the migration and activation of neutrophils. HuMax-IL8, a monoclonal antibody against IL-8, has shown preliminary anti-tumor activity in patients with metastatic or unresectable solid tumors and has been used in Phase I clinical trials. It is safe and well-tolerated [57]. In recent years, a growing body of research has shown that the remodeling of the tumor immune microenvironment is not only associated with changes in immune cell infiltration but is also closely linked to metabolic reprogramming. Abnormal glucose, lipid, and amino acid metabolism collectively contribute to tumor immune evasion by influencing effector T-cell function, promoting the expansion of immunosuppressive cell populations, and regulating the expression of cytokines and immune checkpoint molecules [58, 59]. Meanwhile, recent studies on colorectal cancer also suggest that prognostic indicators integrating inflammation, immunity, and nutritional status offer significant value for risk stratification. For example, models based on inflammation-related hematological markers, as well as composite indices integrating immune and nutritional status, have demonstrated certain predictive capabilities for survival in patients with stage I–III colorectal cancer [60, 61]. These findings suggest that prognostic assessment is gradually shifting from single biomarkers toward multidimensional integrated models. The CAF- and immune evasion-related risk model developed in this study is generally consistent with this research trend and further supports the view that tumor microenvironment remodeling is closely associated with prognostic heterogeneity.

It should be noted that while the prognostic risk model developed in this study demonstrated good predictive performance in the TCGA cohort, its AUC was relatively lower in the GSE39582 external validation cohort, suggesting that the model’s predictive performance varies across different datasets. This variation may be attributed to differences in detection platforms, batch effects, and clinical heterogeneity between cohorts. TCGA-COADREAD is primarily based on RNA-seq data, whereas GSE39582 utilizes the GPL570 chip platform. Differences between these platforms in terms of expression quantification range, probe coverage, and data preprocessing methods may affect the model’s stable performance in external cohorts. Additionally, differences in sample composition, clinical characteristics, and follow-up information across cohorts may further affect the model’s validation results. Nevertheless, the model developed in this study still demonstrated a certain degree of discriminatory power in the independent GEO cohort, suggesting it has some cross-cohort predictive value; however, its generalizability and robustness require further validation in additional independent populations. Despite its limitations, the model demonstrated consistent risk stratification and clear separation of survival outcomes across both datasets, indicating its robustness and biological relevance. By integrating CAFs and immune escape-related genes, this study offers a new perspective on their combined impact on colorectal cancer prognosis. The model is a valuable tool for exploring how CAFs modulate the tumor immune microenvironment and influence clinical outcomes. Future research should focus on incorporating additional clinical variables and validating the model using larger, multicenter, independent cohorts to enhance its clinical applicability and translational potential.

This study has several limitations that warrant consideration. First, since the research was based on retrospective data from the TCGA and GEO public databases, inherent selection and information biases associated with retrospective studies could not be entirely avoided, despite external validation being performed. Second, although experimental validation utilized HPA and single-cell sequencing of CRC FFPE samples and CAF cells, the limited sample size may have affected the reliability of the results for certain genes. Future studies should further investigate the causal mechanisms by which these hub genes regulate immune escape and tumor progression in CAFs through in vivo and in vitro functional assays. These assays should include gene knockout or overexpression experiments and co-culture systems. Understanding these mechanisms is essential for identifying viable therapeutic targets from potential biomarkers.

In conclusion, this study revealed the mechanism by which CAFs in CRC regulate the immune microenvironment through core genes, as determined by multi-omics analysis. Using the expression patterns of key genes, a dual-functional model was constructed to forecast patient prognosis and response to immunotherapy concurrently. This enabled the classification of patients into two subtypes with significantly different survival outcomes. Validation demonstrated the model’s robustness, showing that the high-risk group had a significantly shorter overall survival period. The low-risk group displayed an “immune-activated” phenotype characterized by higher expression of immune checkpoint molecules, increased TMB, and reduced potential for immune escape. This indicates a greater likelihood of responding to immunotherapy. Ultimately, SOX2, SPP1, APOE, CXCL11, and CXCL8 were identified as core hub genes with strong potential as prognostic biomarkers and therapeutic targets.

## Data Availability

No datasets were generated or analysed during the current study.
